# Sensory and biochemical characterization of a snack based on dried pomegranate from two cultivars during shelf-life under modified atmosphere packaging

**DOI:** 10.1016/j.fochx.2026.104742

**Published:** 2026-11-02

**Authors:** Bernardo Pace, Chiara Medoro, Michela Palumbo, Virginia Carbone, Paola Minasi, Marta Cianciabella, Stefano Predieri, Maria Cefola

**Affiliations:** a Institute of Sciences of Food Production, National Research Council of Italy, c/o CS-DAT, Via Michele Protano, Foggia 71121, Italy; b Institute of Bioeconomy, National Research Council of Italy, Via Gobetti 101, Bologna 40129, Italy; c Institute of Food Science, National Research Council of Italy, Via Roma 64, Avellino 83100, Italy

**Keywords:** Wonderful, Acco, Hot-air drying, Ellagitannins, Anthocyanins, TCATA

## Abstract

Hot air drying is an effective method to extend pomegranate arils' shelf-life, even if its effects on both biochemical composition and sensory quality remain insufficiently characterized.

Two pomegranate cultivars, Wonderful (WF) and Acco (AC), were processed by hot-air drying and stored under modified atmosphere packaging for 120 days. A total of 53 phenolic compounds were tentatively identified by UHPLC-MS^n^ and quantified by HPLC-UV/Vis. In fresh arils WF showed a higher total phenolic content than AC (226.62 and 175.10 mg 100 g^−1^ fw, respectively), while in arils after dehydrations (day 0) AC presented double phenolic concentrations compared to WF. Colour stability was higher in WF, whereas AC exhibited greater chromatic degradation. Regarding sensory evaluation, WF arils were less susceptible to aromatic degradation compared to AC. Descriptive Analysis (DA) and Temporal Check-All-That-Apply (TCATA) of WF arils indicated partial sensory stability up to 60 days, followed by a progressive sensory decline in most of the sensory attributes analysed.

## Highlights

• Hot-air drying affects biochemical profile and sensory of dried pomegranate arils
• Wonderful cultivar showed higher total phenolic content in fresh arils
• Acco exhibited higher concentrantion of phenols in dried arils during storage
• Wonderful dried arils were less susceptible to aromatic degradation
• TCATA test of Wonderful dried arils indicated a sensory stability up to 60 days

## 1 Introduction

Pomegranate (*Punica granatum* L.), native to the Middle East, is among the oldest cultivated fruit species and is presently grown in numerous regions worldwide. The fruit is widely recognized as a functional food owing to its high concentration of bioactive compounds, including phenolic acids, flavonoids, vitamins, organic acids, sugars, antioxidants, and mineral elements. These constituents have been associated with several health-promoting effects, particularly the mitigation of oxidative stress and inflammatory processes (Hooks et al., 2021; Li et al., 2015; Masci et al., 2016; Palumbo et al., 2024; Teixeira Da Silva et al., 2013). However, the content and profile of these bioactive compounds are strongly influenced by genetic factors (cultivar), environmental conditions, ripening stage, and both pre- and post-harvest practices (Borochov-Neori et al., 2009; Poyrazoğlu et al., 2002). More than 90% of the total antioxidant activity of pomegranate has been attributed to two major classes of polyphenolic compounds: anthocyanins - primarily delphinidin, cyanidin, and pelargonidin, which are responsible for the characteristic red coloration of the peel and juice - and hydrolysable tannins, including punicalin, pedunculagin, punicalagin, as well as gallagic and ellagic acid glucose esters (Gil et al., 2000; Viuda-Martos et al., 2010).

Arils constitute the edible, fleshy fraction of the fruit and are characterized by a short shelf-life and high perishability (Caleb et al., 2013). However, drying processing could be considered an effective method to extend arils' shelf-life, by reducing moisture content, thereby inhibiting microbial activity (Alaei & Amiri Chayjan, 2015; Dak et al., 2014; Fellows, 2022; Maphosa et al., 2025). Dried pomegranate arils have diverse culinary applications, as they can be consumed as snacks, used as condiments, or incorporated into mixtures with other dried fruits. Several studies have demonstrated that the inclusion of dried fruits in the human diet provides health benefits due to their high concentration of bioactive compounds, including those present in pomegranate arils (Hmid et al., 2017; Cano-Lamadrid et al., 2018; Gyurova & Enikova, 2014; Scrob et al., 2022).

Sensory evaluation performed by trained assessors is a fundamental approach for accurately characterizing product attributes and predicting consumer acceptance. Trained sensory panels have been employed to evaluate pomegranate arils in order to investigate the effects of different drying techniques (Calín-Sánchez et al., 2013; Cano-Lamadrid et al., 2018), to assess textural properties (Szychowski et al., 2015), and to analyse the influence of cultivar and production area prior to consumer testing (Rozzanigo et al., 2021). In other studies, the identification of sensory preference drivers has been carried out directly with consumers using specific methods such as the Check-All-That-Apply (CATA) test (Pons-Gómez et al., 2024) or basic hedonic evaluation (Chater et al., 2018).

The aim of this study was to characterize the biochemical and sensory evolution of dried pomegranate arils obtained from Wonderful and Acco cultivars, packaged under modified atmosphere and stored for up to 120 days, to compare the biochemical and sensory evolution of dried pomegranate arils obtained from Wonderful and Acco cultivars during storage.

Throughout the storage period, biochemical and sensory characterizations were carried out, revealing significant differences between cultivars. Furthermore, in a second experimental phase, a descriptive sensory analysis combined with Temporal Check-All-That-Apply (TCATA) evaluation was performed on the cultivar showing the best overall performance.

## 2 Materials and methods

### 2.1 Materials and chemicals

High-Performance Liquid Chromatography (HPLC) grade acetonitrile and formic acid were obtained from Merck (Darmstadt, Germany). HPLC grade water (18.2 mΩ cm) was prepared using a Millipore Milli-Q purification system (Millipore Corp., Bedford, MA, USA). Gallic acid, vanillic acid, caffeic acid, syringic acid, p-coumaric acid and ferulic acid were purchased from Sigma-Aldrich (St. Louis, MO, USA). Cyanidin-3-O-glucoside chloride (Kuromanin chloride), delphinidin-3-O-glucoside chloride (Myrtillin chloride) and ellagic acid were purchased from Extrasynthese (Genay, France).

For the quality determinations on fresh pomegranate arils, extraction solvents (methanol, ethanol), 2,2-diphenyl-1-picrylhydrazyl (DPPH), 6-hydroxy-2,5,7,8-tetramethylchroman-2-carboxylic acid (Trolox), gallic acid and sodium hydroxide (NaOH) were obtained from Sigma–Aldrich (St. Louis, Mo., USA). Folin-Ciocalteu's phenol reagent was purchased from Merck (Germany).

### 2.2 Fresh pomegranate quality assessment

Pomegranate fruits (*Punica granatum* L.) of Wonderful (WF) and Acco (AC) cultivars, were handpicked at commercial harvest period from a local farm in Foggia (southern Italy). These cultivars were selected because they are among the most commercially relevant pomegranate cultivars in the Mediterranean region and have been extensively studied due to their distinct agronomic, physicochemical, and sensory characteristics. At harvest, fruits with uniform colour and size and without visible external defects were transported to the Postharvest Laboratory at the Institute of Sciences of Food Production of National Research Council (ISPA CNR). Fifteen fruits, for each cultivar, were washed in chlorinated water (1.34 mmol NaOCl L^−1^), rinsed with tap water and drained, and then used for the following quality determinations in triplicate. Whereas the remaining 60 fruits were processed to obtain dried arils as below reported.

Pomegranate juice was obtained using a squeezer (Juicer SZP 25 B2, Silver Crest, Hamburg, Germany). The pH was measured using a pH-Burette 24 (Crison Instruments, Barcelona, Spain), while titratable acidity (TA) was determined by acid–base potentiometric titration with 0.1 mol L^−1^ NaOH up to pH 8.1. Titratable acidity was expressed as percentage (%) of citric acid, considered the predominant organic acid in pomegranate juice.

Total soluble solids (TSS) were measured at room temperature using a digital refractometer (DBR35, XS Instruments, Carpi, Italy), and the results were expressed as °Brix, according to the method described by Palumbo et al. (2024). Antioxidant activity (AA) and total phenols (TP) were carried out using spectrophotometric methods, employing a UV–Vis spectrophotometer (UV-1800, Shimadzu, Kyoto, Japan). Antioxidants were extracted directly from the juice using the DPPH (1,1-diphenyl-2-picrylhydrazyl) assay (Palumbo et al., 2024). The TP content was analysed according to Singleton and Rossi (1965), in which properly diluted pomegranate juice was mixed with distilled water, Folin-Ciocalteu reagent and 200 g L^−1^ sodium carbonate solution. Following 2 h of incubation in darkness, the absorbance was read at 765 nm. The TP content was assessed based on the gallic acid standard curve and expressed as mg of gallic acid per 100 g of fresh weight (mg gallic acid 100 g ^−1^).

In addition, phenolic compounds from 5 g of fresh sample were extracted with 7 mL of acetone as previously described (Palumbo et al., 2024). Briefly, extraction was performed for 10 min on a horizontal shaker in the dark, centrifugation at 4000 rpm for 10 min, and the supernatant was removed. The pellet was suspended in 5 mL of 2% formic acid in acetone and the extraction was carried out as described. The supernatant was removed, the pellet was suspended in 3 mL of acetone/ water 70/30 and the extraction was carried out as described before. This extraction was repeated twice and the supernatants were pooled. The extracts obtained were used for the analysis of phenol compounds as below reported in paragraph 2.3.4 and 2.3.5.

### 2.3 Drying, packaging and storage in modified atmosphere of pomegranate arils

The study was conducted in two experimental phases. In the first phase, arils from each cultivar were pooled, homogenized, randomized, and subjected to hot-air drying. The dried batches were used for colour, a_w_, package headspace composition, phenolic, and preliminary sensory analyses. All measurements were performed on subsamples obtained from the same batch. In the second phase, a separate batch of WF dry arils was produced, using the same processing and storage conditions and was employed for Descriptive Analysis (DA) and Temporal Check-All-That-Apply (TCATA).

A flowchart of the experimental design is reported in Fig. S1.

Before drying, after manual separation, arils from each cultivar were pooled, homogenized, and randomized.

The arils were processed (approximately 60 fruit per cultivar, WF or AC) by hot-air drying without prior pretreatment, carried out in a 12-tray De Luxe B12 dryer (Tauro Essiccatori, Camisano Vicentino, Italy).

This drying method is considered effective for extend arils' shelf-life, by reducing moisture content, and consequently inhibiting microbial activity (Maphosa et al., 2025; Alaei & Amiri Chayjan, 2015; Dak et al., 2014; Fellow, 2022). Samples were dried at temperature of 60 ± 2 °C for about 48 h until reaching a water activity (a_w_) value lower than 0.45 ± 0.02. The obtained dried arils, for each cultivar, were again mixed and randomized prior to packaging and storage to ensure sample homogeneity. For each cultivar, about 50 g of arils were placed in polypropylene/polyamide (PP/PA) bags of 15 × 25 cm (PA 15 μm, PP 60 μm, O_2_ transmission rate 40 cm^3^ m^−2^ d bar, Orved, Musile di Piave, Italy,) and packed in modified atmosphere packaging (MAP) applying 0/0/100 Kpa of O_2_/CO_2_/N_2_ respectively, using a packaging machine (Boxer 50 GAS, Lavezzini, Fiorenzuola d'Arda, Italy) equipped with gas-mixer (KM-100–3 WITT-Gasetechnik, Witten, Germany). Nitrogen was selected because it is an inert gas widely employed for low-moisture products to reduce oxidative reactions affecting pigments, aroma compounds, and phenolics while avoiding package collapse associated with vacuum packaging (Caleb et al., 2012; Miranda et al., 2019).

Samples were stored at 20 ± 1 °C in the dark and analysed at time (T), 0, 30, 60, 90, and 120 d.

Representative images (Fig. S2) of dried arils were acquired at selected storage times (T0, T60, and T120) using the same computer vision system employed for colour measurements (Section 2.3.1). Images were obtained under controlled and standardized illumination conditions to visually document colour evolution during storage (Palumbo et al., 2022).

All determinations were carried out on dried arils, as described below. Three independent replicates were prepared and analysed for each cultivar and storage time.

#### 2.3.1 Headspace analysis, water activity, residual moisture content and colour measurement of dry arils

Initially and at each storage time, the percentage of gas composition inside the bags was measured using a portable headspace gas analyser (CheckPoint O_2_/CO_2_ Dansensor® Mocon, Ringsted, Denmark).

The analysis for determining water activity (a_w_) of samples were conducted on hot-air-dried samples. at each storage time using the water activity meter Humimeter RH2 (Schaller Messtechnik GmbH St. Ruprecht/Raab, Austria).

The residual moisture content, determined according to AOAC Method 934.06 (ISO – International Organization for Standardization, 2023), was calculated according to the following equation:

$$\text{Moisture}\;\left(\%\right)=\frac{\left(W1-W2\right)\cdot 100}{W1.}$$

where W1 was the weight (g) of sample before drying, while W2 was the weight (g) of sample after drying.

The colour parameters of the dried pomegranate arils were measured using a computer vision system (CVS) equipped with an AP3200TPGE RGB digital camera (JAI Ltd., Yokohama, Japan) placed inside a HPB60D photo studio box (HAVOX, Vendôme, France), following the procedure described by Palumbo et al., 2022. Image processing and data analysis were carried out using MATLAB® R2021b (MathWorks Inc., Natick, MA, USA), applying the image analysis procedure described by Gonzalez et al. (2004). Each raw images were segmented from the background using a binary thresholding algorithm, which isolated the foreground and defined the region of interest. The algorithm then separated the image into its three colour components: red, green, and blue (RGB). The RGB data was converted into L*, a*, and b* colour space and Chroma and hue angle (h°) were calculated.

#### 2.3.2 Sensory quality evaluation

A group of six expert assessors conducted an exploratory sensory screening of the two cultivars. This preliminary evaluation was intended solely to support cultivar selection for the subsequent in-depth sensory study and should not be considered a formal descriptive sensory analysis. Given its exploratory purpose, the results were interpreted together with physicochemical and storage-stability data to identify the cultivar showing the most promising overall performance. Accordingly, the sensory findings are presented as preliminary evidence and should be interpreted within the constraints imposed by the limited panel size.

Sensory attributes were selected and evaluated using 5-point rating scale (from 5 = best quality to 1 = worst quality). The assessed parameters included visual appearance, characteristic smell, dislike smell, taste, crunchiness, chewiness, and overall quality. The descriptive definitions associated with each score are reported in Table S1 and were adapted from previously published methodologies (Cano-Lamadrid et al., 2018; Thakur et al., 2023).

The outcomes were employed to identify the cultivar and storage duration most suitable for conducting a detailed sensory evaluation described in section 2.4.

#### 2.3.3 Extraction of phenolic compounds by dried pomegranate arils

For the extraction of phenolic compounds, 1 g of dried arils from each sampling times and from both WF and AC cultivars was rehydrated with 4 mL of water for 1 h prior to extraction. Successively, 7 mL of acetone was added, and extraction was carried out as for fresh samples. All extracts were dried in a rotary evaporator (LaboRota 4000/HB Efficient, Heidolph, Schwabach, Germany) and stored at −20 °C until the use.

#### 2.3.4 UHPLC-MS^n^ analyses

The extracts were reconstituted in water and analysed by ultra-high performance liquid chromatography (UHPLC)-linear ion trap mass spectrometry on an LTQ XL ion-trap mass spectrometer coupled with a UHPLC Ultimate 3000 RS chromatographic system equipped with a Diode Array Detector and Xcalibur® system manager data acquisition software v4.5 (Thermo Fisher Scientific, Bremen, Germany). Chromatographic and mass spectrometric conditions followed those previously reported (Palumbo et al., 2024). Briefly, phenols were separated on a XBridge BEH C18 column (130 Å, 5 mm, 2.1 mm × 150 mm, Waters) at a flow rate of 200 μL min^−1^ using water/acetonitrile/formic acid (96:4:0.1 v/v/v) as solvent A and water/acetonitrile/formic acid (46:54:0.1 v/v/v) as solvent B. After a 2 min hold at 0% solvent B, elution was performed by a linear gradient from 0 to 20% solvent B in 26 min, from 20 to 40% solvent B in 15 min, from 40 to 60% solvent B in 5 min and from 60 to 95% solvent B in 5 min, followed by 10 min of maintenance. The column effluent was split into two using a “T junction” placed after the chromatographic column and analysed “on-line” both by UV–Vis (wavelength range 200–800 nm) and heated electrospray ionization mass spectrometry (HESI/MS). The capillary voltage was set at −27 V, the spray voltage was −4 kV, and the tube lens offset was −87 V in negative ion mode, while, in positive ion mode, the capillary voltage was set at 4 V, the spray voltage was 4 kV, and the tube lens offset was 140 V. The heater temperature was set at 200 °C and the capillary temperature was 275 °C. Mass spectra were recorded from mass-to-charge ratio (*m*/*z*) 100 to 2000 both in negative and positive ionization mode and data were acquired in MS, MS/MS, and MS^n^ scanning modes. Zoom scan analyses were carried out to determine the charge state of molecular ions originating from some of the ellagitannin-based compounds.

#### 2.3.5 HPLC-UV–vis analyses

Quantitative HPLC analyses of main phenolic compounds identified in extracts were conducted on an HP 1100 Series HPLC (Agilent, Palo Alto, CA, USA) equipped with a binary pump and an UV–Vis detector using the previously described conditions (Palumbo et al., 2024). Briefly, individual phenols were separated on a XBridge BEH C18 column (130 Å, 5 mm, 4.6 mm × 150 mm, Waters) at a flow rate of 1 mL min^−1^; the elution program was the same described in the paragraph 2.3.4, while solvent A was water-acetonitrile-formic acid (95:4:1 v/v/v) and solvent B was water-acetonitrile-formic acid (44:55:1 v/v/v). Compounds were detected at 280 nm and at 520 nm anthocyanins. Quantification of phenolic compounds was performed with external calibration curves generated by repeated injections of appropriate standards prepared over a concentration range of 1–50 μg mL^−1^ with five different concentration levels and duplicate injections at each level. Ellagitannins were quantified as ellagic acid equivalents whereas gallotannins as gallic acid equivalents. Anthocyanins quantification was performed with external calibration curves generated by repeated injections of a fixed volume of standard solutions of cyanidin-3-O-glucoside and delphinidin-3-O-glucoside over a concentration range of 0.1–50 μg mL^−1^ with five different concentration levels and duplicated injections at each level. All samples had three replications, and each replicate was measured twice. Phenolic compounds quantified in fresh arils were expressed as mg 100 g^−1^ fresh weight (fw), whereas phenolic compounds in dried arils were expressed as mg 100 g^−1^ dry weight (dw). The use of a dw basis for dried samples was necessary to account for the substantial reduction in moisture content caused by dehydration and to enable meaningful comparisons of phenolic concentrations during storage independently of water loss effects. Fresh arils were instead expressed on a fw basis, which is the standard convention for reporting the composition of fresh fruit products.

### 2.4 Descriptive and temporal (TCATA) sensory evaluation of wonderful dried arils during storage

A second trial, carried out on WF cultivar was realized following the same drying process and packaging condition of the above reported first trial, with the aim to assess the sensory characterization using the TemporalTCATA methodology (Ares et al., 2015) and the descriptive analysis at storage time: T0, T60, and T120.

#### 2.4.1 Panel training and test organization

The evaluations were conducted in the sensory analysis and consumer science laboratory of the Institute of BioEconomy of National Research Council (IBE-CNR), in Bologna, using individual booths equipped with tablets and dedicated data collection software (FIZZ, Biosystemès, Couternon, France). Sessions were conducted in accordance with UNI 8589:1990 (UNI -Ente Nazionale Italiano di Unificazione, 1990) guidelines. Before beginning the study, assessors were briefed on the objectives and signed informed consent forms authorizing the use of anonymized data. Participation was voluntary, and all procedures adhered to current privacy and data protection regulations (GDPR 2016/679).

The arils sample was evaluated three times for each storage time (T0, T60, and T120) resulting in a total of 30 assessments for each time.

A specific training was conducted to ensure consistency, repeatability, and a shared understanding of attribute definitions and intensity assessment. Judges with the described relevant background knowledge, underwent an 8 h training session focussed on the sensory evaluation of industrially produced dried pomegranate arils (Naturzo ®, Segewold, Latvia) to familiarize themself with the specific sensory attributes of the product.

#### 2.4.2 Descriptive analysis

The Descriptive Analysis (DA) was carried out with a panel of 10 trained assessors, each with over 80 h of prior experience in the sensory assessment of various food matrices, including fresh and dried fruits. The overall procedure was in line with ISO 13299:2016 (International, 2023) recommendations for descriptive sensory methodologies, using intensity scales consistent with ISO 8586:2023 (ISO – International Organization for Standardization, 2023).

A set of 26 sensory descriptors was defined based on existing literature (Mayuoni Kirshenbaum et al., 2016; Sinesio et al., 2012) and complemented with additional terms to ensure an adequate descriptive vocabulary for DA. All attributes were evaluated using a 9-point intensity scale, ranging from “no perception” to “maximum perceivable intensity.” Water was supplied for palate cleansing between tastings.

#### 2.4.3 Temporal check-all-that-apply

Temporal Check-All-That-Apply (TCATA) was selected because it allows assessors to simultaneously report multiple sensory attributes over time, providing a more comprehensive description of complex products than methods such as Temporal Dominance of Sensations (TDS), which records only the dominant sensation at a given moment (Ares et al., 2015; Castura et al., 2016). Although dynamic sensory methods have been increasingly applied to fruit-based products, their application to dried pomegranate arils remains limited. Therefore, the present study contributes novel information regarding the temporal evolution of flavour and texture attributes during storage.

Wonderful was chosen for the dynamic sensory evaluation since it represents the most commercially relevant and widely studied pomegranate cultivar worldwide (Caleb et al., 2012; Holland et al., 2009; Stover and Mercure, 2007).

The TCATA method (Ares et al., 2015) was used to capture the dynamic evolution of panelists' sensory perceptions. This approach extends traditional check-all-that-apply (CATA) questions by allowing assessors to indicate, at any moment during the evaluation, all sensory attributes they perceive. Ten panelists, already trained on arils' sensory analysis for the previous DA test, were further instructed (2 sessions of training, 60 min each) to recognize and scale all sensory attributes relevant to the target samples across the different sensory modalities and perform the TCATA test in duplicate. Panelists were instructed to put 10 arils in their mouths through a teaspoon and concurrently clicking a start button. Panelists had to select, throughout the assessment (60 s), the attributes that clearly describe their sensations at each time point and deselect them as soon as those sensations fade from a list. The attribute list, selected from the previous DA test, included five flavour attributes (pomegranate, red fruits, flower, raisin and winey); two taste attributes (acid and sweet); and eight texture attributes (firmness, crunchiness, graininess, mouth coating, moisture, astringency, tingling, and numbing). TCATA was performed as an in-mouth tasting test; therefore, visual and aroma attributes were not included. The overall flavour attribute was also excluded to encourage assessors to better focus on specific sensory traits.

### 2.5 Statistical analysis

All data acquired on fresh samples were analysed running a one-way ANOVA, in order to evaluate the effect of the cultivar. Instead, for physical data measured on dry samples, a two-way ANOVA was carried out in order to evaluate the effect of cultivar (WF and AC), storage (0, 30, 60, 90, 120 d) and their interaction. Statistical analyses were carried out using the software Statgraphics Centurion (version 18.1.12, Warrenton, VA, USA) and Tukey was used as posthoc test (*P* ≤ 0.05). Because each cultivar was represented by a single harvest and a single drying batch, the statistical analysis was intended to describe differences among the sampled batches and storage times under the experimental conditions adopted, rather than to infer cultivar effects at the population level.

In the second trial, sensory data were analysed using the software R (version 4.3.1; R Core Team, 2023) in combination with the SensoMineR package (version 1.2), specifically designed for sensory analysis. A one-way ANOVA was performed on the DA intensity ratings, and Tukey's post-hoc test was subsequently applied to assess significant pairwise differences among samples. Statistical significance was defined at *P* ≤ 0.05. The mean intensity scores obtained from the descriptive analysis were then used to generate a spider plot representing the sensory profiles of the arils sample. For TCATA data the Product Trajectories Over Time approach was implemented (Castura et al., 2016). Correspondence analysis was used to summarize relationships between products and attributes, starting from a contingency table of CATA counts with products in rows and attributes in columns with one such contingency table per time slice (10 s). Product trajectories for the sequence of times points were smoothed along each dimension independently using the R function loess (Cleveland et al., 1992). TCATA assesors repeatability and replication were measured using city block distance as indicated by Catura et al., 2016.

## 3 Results and discussion

### 3.1 Physicochemical quality attributes of fresh pomegranate juice from wonderful and Acco cultivars

The main quality parameters of fresh arils from both cultivars were evaluated and results are reported in Table S2. Overall, the arils showed physicochemical and bioactive characteristics consistent with those reported in the literature for commercially mature pomegranate fruits. In detail, the pH values recorded for WF (3.27 ± 0.32), and AC (2.90 ± 0.02) fell within the typical range described for pomegranate arils, with no statistical differences among the cultivars (Nikdel et al., 2016; Rahimi et al., 2017). Both TSS content and TA showed higher values in WF than AC, agreed with previously reported data for both cultivars and confirming genotype-related differences in perceived sweetness and in organic acid composition (Palumbo et al., 2024; Passafiume et al., 2019; Tarantino et al., 2022). As regards the chemical quality parameters, the AA and TP of WF arils was respectively 1.2 and 1.3-fold higher than that measured in AC (Table S2). These results are consistent with the study conducted by Tarantino et al. (2022) on several pomegranate genotypes, who recorded higher performances in antioxidant activity and total phenols in Wonderful. Generally, cultivars with the highest phenols content also have higher antioxidant activity compared to those characterized by lowest phenols (Gil et al., 2000; Melgarejo-Sánchez et al., 2015).

#### 3.1.1 Phenolic profile of acetone extracts obtained from fresh pomegranate arils

Fresh pomegranate arils extracts were prepared from two different cultivars, WF and AC, and analysed by UHPLC-MS^n^ and HPLC-UV–Vis. As an example, the HPLC-UV–Vis chromatograms of the fresh and dry (T 0) pomegranate arils extract of WF cultivar are shown in Fig. S3. A total of 53 phenolic compounds were tentatively identified in the different extracts, which include anthocyanins, hydrolysable tannins, hydroxycinnamic acids, vanillic acid derivatives, organic acids and neolignans, as reported in Table S3. These results are consistent with those previously published on the extract of fresh pomegranate arils of the cultivar WF (Palumbo et al., 2024). Regarding anthocyanins, all extracts from the different samples of fresh pomegranate arils contained delphinidin 3,5-diglucoside (peak 1), delphinidin 3-glucoside (peak 3), cyanidin 3,5-diglucoside (peak 2), cyanidin 3-glucoside (peak 4), cyanidin 3-pentoside (peaks 6 and 7) and pelargonidin 3-glucoside (peak 5) (Fig. S3, Table S3), in accordance with those already reported in our previous paper (Palumbo et al., 2024). Moreover, in the fresh pomegranate arils extract of AC cultivar coumaric acid hexoside (peak 30) was also found (Table S3). The presence of this phenolic compound was already reported in previous studies on pomegranate fruit (Brighenti et al., 2017; Hassanen et al., 2019; Sentandreu et al., 2013). Results of the ANOVA performed on data obtained by quantitative analysis of individual and total phenolic compounds in extracts of fresh intact arils belonging to both WF and AC cultivars are reported in Table S4. Moreover, in Fig. 1 the concentration of the total phenolic compounds and of the most abundant phenolic compounds identified in fresh WF and AC pomegranate samples are reported. The level of total phenolic content varied significantly (*P* ≤ 0.05) between the WF and AC, ranging from 226.62 to 175.10 mg 100 g^−1^ fw, respectively (Fig. 1; Table S4). Moreover, the content of delphinidin 3,5-diglucoside (peak 1), delphinidin 3-glucoside (peak 3), galloyl-hexoside (peak 10), oenotheinB isomer 1 (peak 28), vanillic acid derivative 1 (peak 31), vanillic acid derivative 2 (peak 32) were significantly higher in fresh WF extracts than in fresh AC ones (Fig. 1; Table S4). Overall, fresh pomegranate arils extract of WF cultivar showed a higher anthocyanin content (25.57 mg 100 g^−1^ fw) than the AC one (11.00 mg 100 g^−1^ fw) and this result is in accordance with previous studies on different pomegranate cultivars (Montefusco et al., 2021; Tozzi et al., 2020). On the contrary, the HHDP-valoneoyl-glucoside (peak 25) content is significantly higher in the fresh AC extracts. In addition, the area of the HPLC peak containing digalloyl-HHDP-hexoside (Pedunculagin II) isomer 1 and HHDP-gallagyl-hexoside (Punicalagin) isomer 3 (peak 29), and that of the peak containing digalloyl-HHDP-hexoside (Pedunculagin II) isomer 2 and Ellagitannin 3 (peak 37) were found to be higher in fresh WF extracts than in fresh AC ones, thus suggesting that the amount of molecules present in these peaks, measured as ellagic acid equivalents, was significantly higher in the first extract. Instead, the area of the HPLC peak containing eucarpanin T1 isomer 2, ellagic acid-deoxyhexoside and ellagic acid-pentoside (peak 44) was found to be higher in fresh AC extract, demonstrating that the amount of molecules present in this peak, measured as ellagic acid equivalents, was significantly more abundant in AC than in WF (Fig. 1).

**Fig. 1:**
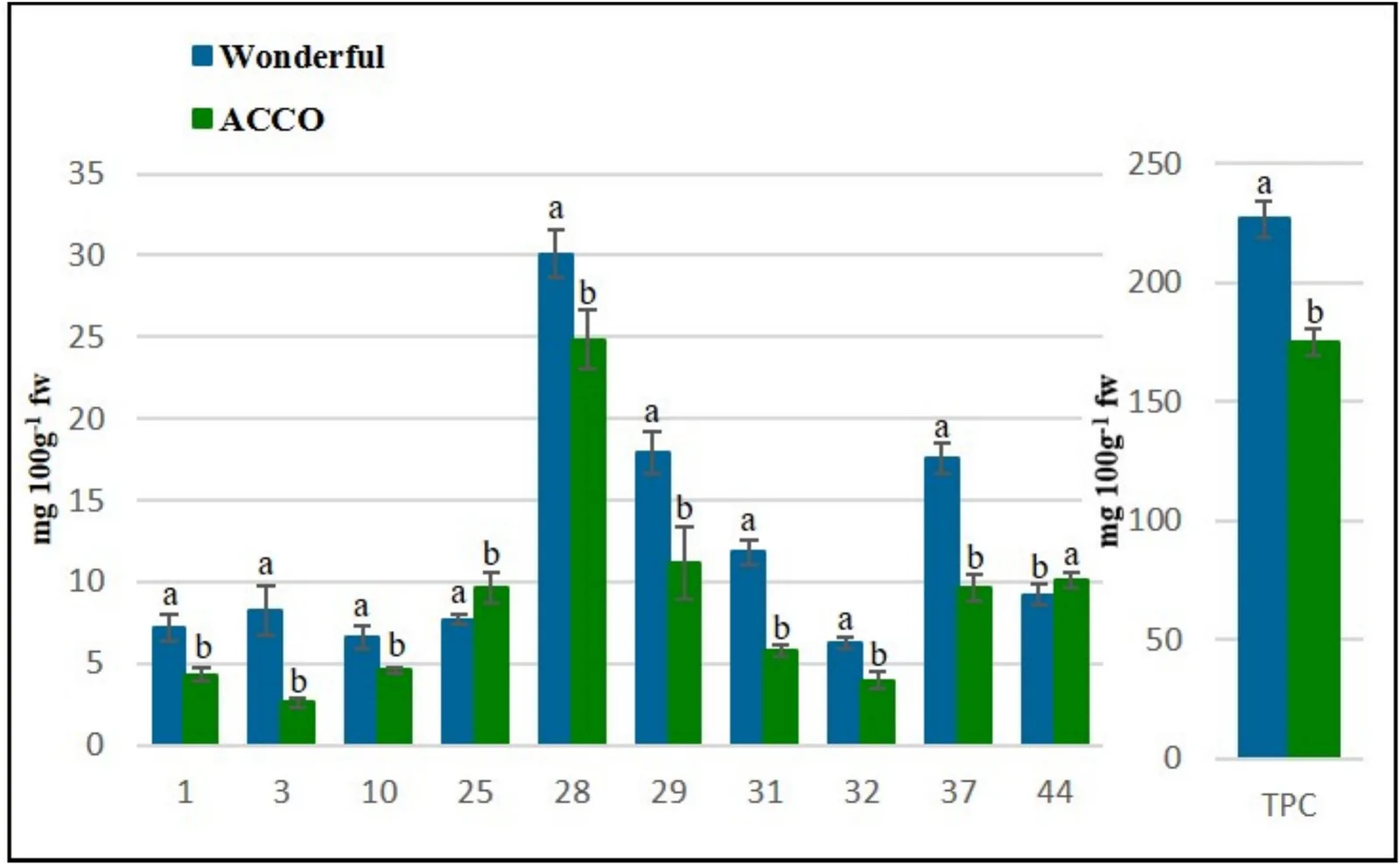
Concentration of the total phenolic compounds and of the most abundant phenolic compounds identified in fresh Wonderful One and Acco pomegranate samples. Different letters represent significant differences (*P* ≤ 0.05). Each bar represents the mean and standard deviation (*n* = 3). The results of the analysis of all phenolic compounds are reported in the Table S4. fw, fresh weight; 1, Delphinidin 3,5-diglucoside; 3, Delphinidin 3-glucoside; 10, Galloyl-hexoside; 25, HHDP-valoneoyl-glucoside; 28, Oenothein B isomer 1; 29, Digalloyl-HHDP-hexoside (Pedunculagin II) isomer 1 + HHDP-gallagyl-hexoside (Punicalagin) isomer 3; 31, Vanillic acid derivative 1; 32, Vanillic acid derivative 2; 37, Digalloyl-HHDP-hexoside (Pedunculagin II) isomer 2 + Ellagitannin 3; 44, Eucarpanin T1 isomer 2 + Ellagic acid-deoxyhexoside + Ellagic acid-pentoside; TPC, Total Phenolic Contents.

It should be noted that total phenolic content determined by the Folin-Ciocalteu assay on juice and total phenolic compounds quantified by HPLC in aril extracts provide complementary rather than equivalent information. Differences between these values are expected because of the distinct sample matrices, extraction procedures, and analytical methodologies employed.

The results reported in this section derive from acetone extracts of whole fresh arils, whereas the parameters presented in Section 3.1 were measured directly on juice samples; therefore, the two datasets are not directly comparable.

### 3.2 Quality evolution of dried pomegranate arils during storage

#### 3.2.1 Physical stability of dried arils during storage

Headspace gas composition was monitored throughout storage. Oxygen concentrations remained below 3% up to T60, followed by a progressive increase to approximately 5% at T120, consistent with the known permeability characteristics of PP/PA packaging under nitrogen flush (Miranda et al., 2019). While, CO_2_ levels remained negligible throughout storage, reflecting the low metabolic activity of the dried matrix. No significant differences in headspace composition were observed between cultivars (data not shown). The colour evolution of dried pomegranate arils from both cultivars (WF and AC) was measured as objective indicators of colour stability and pigment integrity in dehydrated fruit matrices.

To facilitate the interpretation of instrumental colour measurements, representative images of dried arils from both cultivars at T0, T60, and T120 are shown in Fig. S2. The visual appearance of the samples is consistent with the colourimetric results, highlighting the greater colour stability of WF and the more pronounced colour changes observed in AC during storage. Results of the two way ANOVA carried out on the quality parameters of dry arils are reported in Table S5. All the colour parameters showed statistical differences among the factors. In detail, L* and h° were significantly influenced by the cultivars, with lower values in WF cultivar than AC; while the cultivar and the interaction of factors statistically affected a*, b* and Chroma parameters. The significant cultivar × storage interaction observed for a*, b*, and Chroma indicates that colour changes during storage were cultivar-dependent. While WF arils showed relatively small fluctuations in these parameters, AC arils exhibited a more pronounced downward trend over storage (Fig. 2). However, because the main effect of storage time was not significant, these trends should be interpreted as differences in cultivar response to storage rather than as evidence of a general storage-induced colour deterioration.

**Fig. 2:**
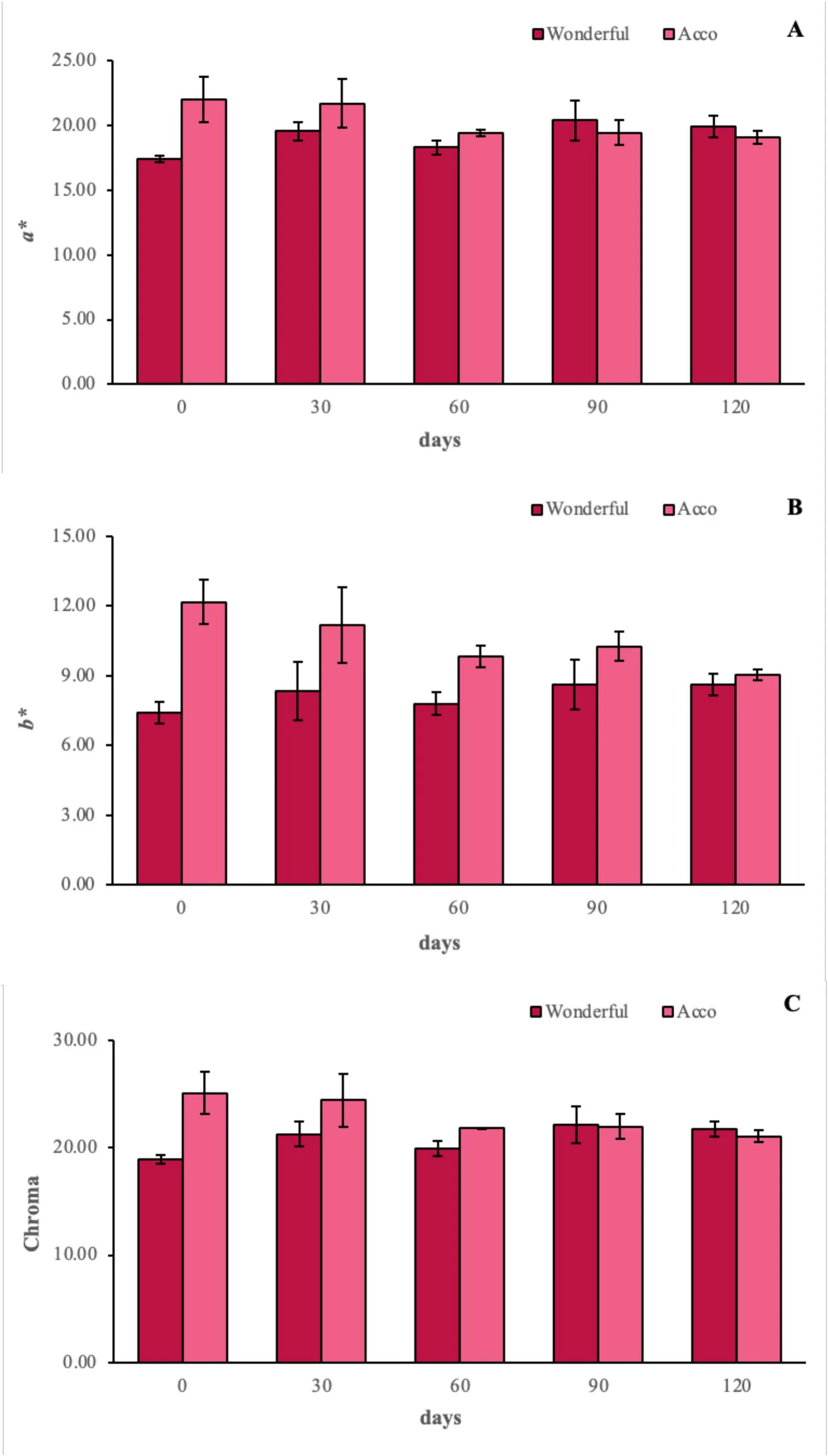
Changes in a* (A), b* (B), and Chroma (C) parameters of dried arils of Wonderful and Acco pomegranate cultivars during 120 days of storage at 20 ± 1 °C under modified atmosphere packaging (100 kPa N₂). Bars represent means ± standard deviation (*n* = 3).

Although significant variations were observed for AC dried arils during storage, they did not affect a marked loss of colour intensity, thanks to the high values of h° which assess a reddish colour of dried arils.

These results on colour parameters suggest that the anthocyanin fraction in WF arils remained relatively stable during storage, likely due to higher antioxidant capacity and more stable pigment structures, as previously reported (Mphahlele et al., 2014; Tzulker et al., 2007). On the contrary, in AC cultivar changes in some colour traits along the storage suggest more evident shifts in colour tone, likely associated with anthocyanin degradation and oxidative processes occurring during storage (Pathare et al., 2013; Patras et al., 2010). These results suggest lower pigment stability in AC arils, likely associated with quantitative differences in anthocyanin and phenolic content, while their overall composition appears substantially similar.

Both cultivars maintained stable L* values throughout storage (Table S5), indicating limited browning phenomena and good structural preservation of the dried matrix, conditions typically associated with extended shelf stability in dehydrated products (Sablani et al., 2011). To conclude, WF exhibited better colour retention and a smaller decline in chromatic intensity than AC, making it more suitable for preserving the visual quality of dried pomegranate arils during storage.

As regards a_w_, results obtained from the multifactor ANOVA showed that the two factors alone (cultivar and storage time) affected this physical trait (Table S5).

At the end of the hot-air drying process, residual moisture content, determined according to AOAC Method 934.06, was 11.0 ± 0.4% and 9.8 ± 0.3% in WF and AC, respectively. Howewer the a_w_ remained well below the critical threshold of 0.60, conditions unfavorable for the growth of most spoilage and pathogenic microorganisms Significant differences were observed between cultivars, with dried arils of cv WF exibiting a_w_ value 1.2-fold higher values than those of AC (0.45 vs 0.37). During storage, slight increase of a_w_ was observed, although values remained within the range generally considered acceptabe under the adopted packaging conditions (Table S5). These values are consistent with those reported in the literature for dried pomegranate arils, where a_w_ values typically range between 0.40 and 0.55 and are associated to conditions considered unfavorable for the growth of most spoilage and pathogenic microorganisms (Gölükcü, 2015; Thakur et al., 2020).

#### 3.2.2 Sensory evolution of wonderful and Acco dried arils during storage

A preliminary sensory evaluation was performed as an exploratory screening step to assess the effect of cultivars, storage time and their interaction on the primary sensory attributes of dried pomegranate arils (Table S6). As the evaluation was conducted by six expert assessors, the results were used primarily to support cultivar selection and were interpreted together with physicochemical data. The cultivar selected underwent a comprehensive sensory characterization employing DA and TCATA sensory analysis.

Overall, the sensory profile of dried pomegranate arils remained relatively stable during storage, with statistically significant changes restricted to a limited number of attributes and showing cultivar-dependent patterns.

In detail, the multifactor ANOVA showed that Characteristic smell was significantly affected by cultivar and by the cultivar × storage interaction, whereas the main effect of storage time was not significant. Dislike smell was significantly affected by storage time and by the cultivar × storage interaction, whereas the main effect of cultivar was not significant Taste was significantly affected by cultivar and by the cultivar × storage interaction, whereas the main effect of storage time was not significant. Finally, visual appearance, chewiness and overall quality were influenced by no factor (Table S6).

In cultivar WF, looking at the Fig. 3, sensory stability was maintained for characteristic smell, dislike smell and taste throughout the storage period. On the contrary, in cultivar AC was exhibited higher deterioration primarily in aroma- and taste-related attributes compared to WF samples. Indeed, characteristic smell and dislike smell intensity decreased significantly over time, reflecting a progressive loss of the typical fruity volatile profile and the development of off-odours. A parallel deterioration trend was observed for taste-related attributes (Fig. 3). Cultivar differences were recorded in crunchiness, which showed higher values in WF dried arils compared to AC ones (Table S6). Moreover, this parameter declined significantly at 90 and 120 days, suggesting progressive structural modifications of the dried aril matrix. These changes are consistent with moisture redistribution and cell wall alterations reported in dehydrated fruit tissues during storage which cause a gradual softening process (Krokida et al., 2001; Vega-Gálvez et al., 2009).

**Fig. 3:**
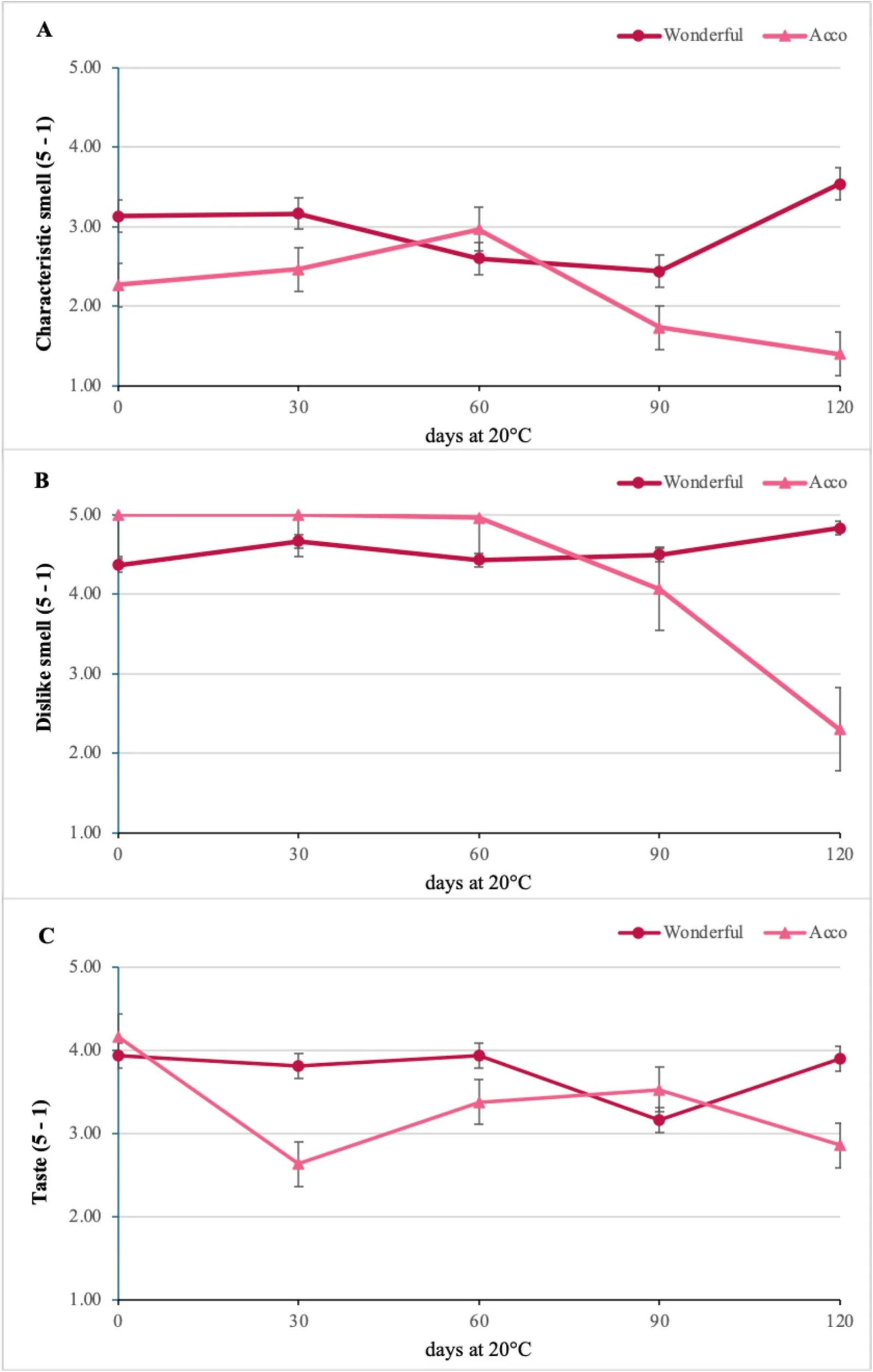
Changes in characteristic smell (A), dislike smell (B), and taste (C) sensory parameters of dried arils of Wonderful and Acco pomegranate cultivars during 120 days of storage at 20 ± 1 °C under modified atmosphere packaging (100 kPa N₂). A 5-point rating scale was used for the evaluation, where 1 = worst quality, 5 = best quality. Values represent means ± standard deviation (*n* = 6).

Taken together, these findings suggest potentially different degradation patterns between the WF and AC batches. AC was predominantly affected by aromatic, flavour and textural deterioration. The aroma and taste deterioration observed in AC may be associated with biochemical alterations commonly reported in dried fruit products during storage, including lipid oxidation and non-enzymatic browning processes (Maskan, 2001; Ratti, 2001); however, these mechanisms were not directly investigated in the present study and therefore remain speculative

The relatively lower susceptibility of WF to aromatic degradation may be related to cultivar-dependent compositional differences; however, because lipid composition, reducing sugars, volatile compounds and oxidation products were not measured, this hypothesis requires further experimental verification.

#### 3.2.3 Phenolic composition and evolution during storage

Extracts from dry arils at each sampling times from both WF and AC cultivars were analysed by UHPLC-MS^n^, and individual and total phenolic contents were measured by HPLC-UV/vis. The qualitative phenolic compounds profiles of both cultivars were not largely affected by drying process, as showed in Table S3, except for extract from dry pomegranate arils of the cultivar AC where cyanidin 3-pentoside (peaks 6 and 7) and pelargonidin 3-glucoside (peak 5) (Table S3) were not detected. As reported before, fresh pomegranate arils extract of AC cultivar showed a lower anthocyanin content than the WF. Jaiswal et al. (2010) highlighted in a study to evaluate the effect of drying on pomegranate aril that drying reduced the amount of anthocyanins.

Moreover, four phenolic compounds, syringic acid-O-hexoside (peak 20), coumaric acid hexoside (peak 30), ferulic acid hexoside (peak 33) and medioresinol (peak 34), were found only in the dry pomegranate arils extract of the WF cultivar (Table S3). The presence of these phenolic compounds was already reported in previous studies on pomegranate fruit (Di Stefano et al., 2019; Hassanen et al., 2019; Sentandreu et al., 2013; Wu & Tian, 2017). Moreover, HHDP-gallagyl-hexoside (Punicalagin) isomer 1 (peak 14) was found only in the dry pomegranate arils extract of the AC cultivar, while Eucarpanin T1 isomer 3 (peak 46) was found in the dry pomegranate arils extract of AC cultivar (but not in the dry pomegranate arils extract of WF cultivar), although it was present in the fresh extracts of both cultivars.

The effect of cultivar, storage time (0, 30, 60, 90 and 120 d) and the interaction of these two factors on the quantitative profile of phenolic compounds in dried arils were determined and results revealed that most of these compounds were significantly affected by the interaction of both cultivar and storage time (Table 1 and Fig. 4). Fig. 4 reported the effects of the interaction of cultivar and storage time on the concentration of the total phenolic compounds and of the most abundant phenolic compounds identified in dried WF and AC pomegranate samples. Statistical analyses showed that the concentrations of total phenolic compounds were significantly affected by interaction of both factors. The highest total phenolic content was consistently observed in the dried arils of AC cultivar, with values of 792.55, 717.10, 730.81, 685.61, and 648.25 mg·100 g^−1^ dw at T0, T30, T60, T90, and T120, respectively, showing an overall decline with a small increase at T60. In contrast, the dried arils of WF cultivar exhibited lower phenolic concentrations, amounting to 409.31, 543.83, 487.20, 443.41, and 484.29 mg·100 g^−1^ dw at the corresponding storage times. (Fig. 4, Table 1), with the content of total phenolic compounds increasing from T0 to T30 then decreasing at T60 and remaining almost similar up to T120. In the fresh extracts, however, we observed that the level of total phenolic content was significantly higher in the extract of fresh arils of WF cultivar than in that of fresh arils of AC cultivar. The cultivar affected the concentration of phenolic compounds in the dried WF and AC samples, as reported in Table 2, where the high concentration of HHDP-gallagyl-hexoside (Punicalagin) isomer 1 (peak 14) (229.98 mg100g^−1^ dw) present only in the dry pomegranate arils extract of the AC cultivar, as seen before, and the significantly higher concentration of some compounds in dried AC pomegranate samples than in the WF ones, are highlighted., The concentration of HHDP-gallagyl-hexoside (Punicalagin) isomer 2 (Peak 21) was significantly higher in dry AC extracts than in dry WF ones (22.37 and 13.37 mg100g^−1^ dw, respectively) (Table 2). In addition, also the area of the HPLC peak containing digalloyl-HHDP-hexoside (Pedunculagin II) isomer 1 and HHDP-gallagyl-hexoside (Punicalagin) isomer 3 (peak 29), was found to be higher in dry AC extracts than in dry WF ones, demonstrating that the amount of molecules present in this peak, measured as ellagic acid equivalents, was significantly more abundant in dried arils of AC than in dried arils of WF (Table 2), while eucarpanin T1 isomer 3 (peak 46) was found only in the dry pomegranate arils extract of AC cultivar, as seen before (Table 2). It has been demonstrated that drying methods caused degradation in bioactive compounds of dried pomegranate arils (Maphosa et al., 2025; Ozay-Arancioglu et al., 2022). In the present study, the impact of drying on phenolic composition appeared to be cultivar-dependent. Despite its lower phenolic content in fresh arils, AC retained higher concentrations of total phenolics and several individual ellagitannins after drying than WF.The interaction of cultivars and storage time also affected the concentration of oenothein B isomer 1 (Peak 28) and eucalbanin B isomer 2 (Peak 42) which are both present in higher concentration in dry AC extracts at all storage times than in those of WF (Fig. 4). In the dried arils of WF the content of oenothein B isomer 1 (Peak 28) increased during the first 30 days of storage, from 44.74 (T 0) to 59.58 mg100g^−1^ dw, decreased both at 60 and 90 d (49.22 and 45.60 mg100g^−1^ dw, respectively), and then increased at 120 days (54.22 mg100g^−1^ dw) (Fig. 4 and Table 1). Instead, in the dried arils of cultivar AC, the content of oenothein B isomer 1 (Peak 28) decreased throughout the storage time (75.78; 74.49; 70.62; 61.63 and 57.86 mg100g^−1^ dw at T0, T30, T60, T90 and T120 respectively) (Fig. 4 and Table 1). In the dried arils of WF also the eucalbanin B isomer 2 (Peak 42) content increased after 30 days of storage (from 16.22 at time 0 to 25.08 mg100g^−1^ dw), decreased both at T60 and T90 d (24.31 and 21.20 mg100g^−1^ dw, respectively), and then increased at T120 (24.96 mg100g^−1^ dw) (Fig. 4 and Table 1). In contrast, in the dried arils of AC cultivar this compound showed a fluctuating trend during storage, with a 22% increase at T60 compared to the mean value of 36.72 ± 0.60 recorded across the other storage periods. (Fig. 4 and Table 1). The concentration of galloyl-HHDP-gluconic acid (Lagerstannin C) isomer 2 (Peak 15) was also influenced by the interaction of cultivar and storage time, resulting for all storage times higher in the dry extracts of the WF cultivar than in those of the AC cultivar at all storage times (Fig. 4). In the dried arils of WF the galloyl-HHDP-gluconic acid (Lagerstannin C) isomer 2 (Peak 15) content increased by 42% after 30 d of storage (from 76.45 at time 0 to 108.50 mg100g^−1^ dw), decreased by 24% at T60 (82.26 mg100g^−1^ dw), increased by 15.6% at T90 (95.09 mg100g^−1^ dw) and then decreased by 11.7% at T120 (83.97 mg100g^−1^ dw) (Fig. 4 and Table 1). Instead, in the dried arils of cultivar AC the content of galloyl-HHDP-gluconic acid (Lagerstannin C) isomer 2 (Peak 15) decreased both at 30 and 60 days of storage (from 10.55 at time 0 to 8.67 and 7.99 mg100g^−1^ dw at 30 and 60 days, respectively), increased at 90 days (9.40 mg100g^−1^ dw) and decreased at 120 days (7.24 mg100g^−1^ dw) (Figure 4 and Table 1). Changes in the nature and quantity of hydrolyzable tannins during the processing and storage of plant-based food matrices are well documented (Vargas-Ramella et al., 2026); however, understanding the biochemical processes underlying the changes we observed during the storage of dried arils from both the WF and AC cultivars would require further study. Results of the two-way ANOVA performed on data obtained by quantitative analysis of total and of all individual polyphenols in extracts of dry pomegranate arils of WF and AC cultivars stored for 0, 30, 60, 90 and 120 d are reported in Table 1.

**Table 1:** Effects of the interaction of cultivar and storage time (0, 30, 60, 90 and 120 days) on the concentration of phenolic compounds (mg 100 g^−1^ dw) identified in dried Wonderful and Acco pomegranate samples. Values with different letters are significantly different among cultivars and storage time at *p* ≤ 0.05.

	**Phenolic compounds**	**Wonderful**										**Acco**									
		0d		30d		60d		90d		120d		0d		30d		60d		90d		120d	
**1**	Delphinidin 3,5-diglucoside	1.70	d	3.70	a	3.05	b	2.55	c	2.38	c	1.77	d	1.07	e	1.16	e	0.80	e	0.82	e
**2**	Cyanidin 3,5-diglucoside	1.83	d	3.48	a	2.92	b	2.52	c	2.27	c	1.40	e	0.88	f	0.99	f	0.69	f	0.70	f
**3**	Delphinidin 3-glucoside	2.63	d	5.79	a	4.54	b	3.43	c	3.96	c	1.29	e	0.95	e	1.37	e	0.67	e	0.75	e
**4**	Cyanidin 3-glucoside	2.73	d	5.07	a	4.05	b	3.17	cd	3.49	c	0.84	e	0.66	e	0.76	e	0.48	e	0.49	e
**5**	Pelargonidin 3-glucoside	0.27	c	0.50	a	0.39	b	0.30	c	0.35	b	nd		nd		0.10	d	nd		nd	
**6**	Cyanidin 3-pentoside	0.14	e	0.30	a	0.25	b	0.18	d	0.21	c	nd		nd		0.10	f	nd		nd	
**7**	Cyanidin 3-pentoside	0.06	d	0.16	b	0.13	bc	0.11	c	0.22	a	nd		nd		0.15	bc	nd		nd	
**10**	Galloyl-hexoside	16.98	b	23.98	a	22.75	a	17.57	b	21.68	a	21.67	a	21.75	a	18.27	b	17.03	b	17.39	b
**11**	Galloyl-HHDP-gluconic acid (Lagerstannin C) isomer 1 + Galloyl-HHDP-hexoside + Unknown	9.80	e	11.48	bcde	11.55	bde	13.15	bcde	11.24	bde	14.20	abcd	10.93	bde	15.38	ac	15.40	abc	17.80	a
**12**	Ellagitannin 1 + Unknown	15.18	ce	19.39	bd	19.29	bd	18.09	d	18.13	cd	20.40	bd	24.48	a	14.48	e	22.32	ab	12.12	e
**15**	Galloyl-HHDP-gluconic acid (Lagerstannin C) isomer 2	76.45	c	108.50	a	82.26	c	95.09	b	83.97	c	10.55	d	8.67	d	7.99	d	9.40	d	7.24	d
**17**	Ellagitannin 2	4.48	b	5.30	ab	6.11	ab	6.04	ab	6.49	ab	7.35	a	6.34	ab	5.73	ab	6.74	ab	5.80	ab
**19**	Caffeic acid hexoside	1.95	d	2.77	cd	2.68	cd	2.47	d	2.94	cd	4.29	b	3.39	bc	7.37	a	4.15	b	3.23	bcd
**22**	Vanillic acid glucoside	7.46	b	10.75	a	8.11	b	7.66	b	7.90	b	nd		nd		nd		nd		nd	
**23**	Caffeic acid hexoside	3.74	e	5.60	cde	5.62	de	4.86	de	7.55	bcd	9.53	ab	11.09	a	8.42	abc	11.37	a	9.34	ab
**25**	HHDP-valoneoyl-glucoside	6.98	abc	8.18	ab	6.44	bc	6.02	bc	4.89	c	8.25	ab	6.55	bc	7.10	abc	9.01	a	5.93	bcd
**26**	Ellagitannin (Camptothin A)	8.81	cd	9.41	cd	8.68	cd	7.44	cd	6.64	cd	13.16	ab	13.94	ab	15.12	a	6.07	d	10.95	bc
**27**	HHDP- hexoside	2.36	c	3.38	ab	2.99	b	3.47	ab	3.87	a	nd		nd		nd		nd		nd	
**28**	Oenothein B isomer 1	44.74	e	59.58	c	49.22	de	45.60	e	54.22	cde	75.78	a	74.49	a	70.62	ab	61.63	bc	57.86	cd
**30**	Coumaric acid hexoside	2.25	c	6.19	a	4.57	b	3.82	b	4.45	b	nd		nd		nd		nd		nd	
**32**	Vanillic acid derivative 2	13.96	bc	15.12	ab	15.04	ab	11.60	cd	12.85	bc	17.76	a	14.25	bc	13.08	bc	13.08	bc	9.48	d
**33**	Ferulic acid hexoside	3.75	d	10.14	a	7.78	b	5.53	c	4.82	c	nd		nd		nd		nd		nd	
**35**	Tri-HHDP-hexoside + Oenothein B isomer 2	nd		nd		nd		nd		nd		18.90	a	13.53	b	14.59	b	15.19	b	14.72	b
**36**	Tri-HHDP-hexoside + Oenothein B isomer 3	8.95	c	9.73	c	10.43	c	8.23	c	14.29	b	13.95	b	15.47	ab	17.51	a	16.67	ab	16.63	ab
**37**	Digalloyl-HHDP-hexoside (Pedunculagin II) isomer 2 + Ellagitannin 3	17.54	bcd	17.73	bc	15.71	bcd	15.15	cd	18.66	b	24.47	a	14.47	d	23.98	a	17.12	bcd	15.29	cd
**39**	Ferulic acid-C-hexoside derivative	3.46	c	4.55	a	3.79	bc	3.77	bc	4.32	ab	nd		nd		nd		nd		nd	
**41**	Eucalbanin B isomer 1	12.42	d	14.61	d	12.52	d	11.63	d	14.10	d	24.56	ab	19.51	c	25.36	a	20.87	bc	20.72	bc
**42**	Eucalbanin B isomer 2	16.22	d	25.08	c	24.31	c	21.20	cd	24.96	c	37.31	b	36.76	b	47.10	a	35.89	b	36.95	b
**43**	Eucarpanin T1 isomer 1 + Galloyl-HHDP-DHHDP-hexoside (Granatin B) + Galloyl-chebuloyl-HHDP-glucose (Chebulagic acid) + Coumaric acid derivative	20.48	de	28.87	a	24.62	bc	21.17	cd	28.16	ab	20.62	de	17.06	ef	15.28	fg	12.28	gh	8.70	h
**44**	Eucarpanin T1 isomer 2 + Ellagic acid-deoxyhexoside + Ellagic acid-pentoside	4.54	e	7.59	d	6.48	de	6.59	de	7.38	d	14.88	c	14.48	c	21.78	a	15.83	c	18.98	b
**49**	Guaiacyl(8–5)ferulic acid hexoside	2.05	b	2.80	a	2.04	b	2.07	b	2.72	a	nd		nd		nd		nd		nd	
	Total Phenolic Contents	409.31	d	543.83	c	487.20	cd	443.41	d	484.29	cd	792.55	a	717.10	ab	730.81	ab	685.61	b	648.25	b

Abbreviations used: HHDP, hexahydroxydiphenoyl; DHHDP, dehydrohexahydroxydiphenoyl; nd, not detected.

**Fig. 4:**
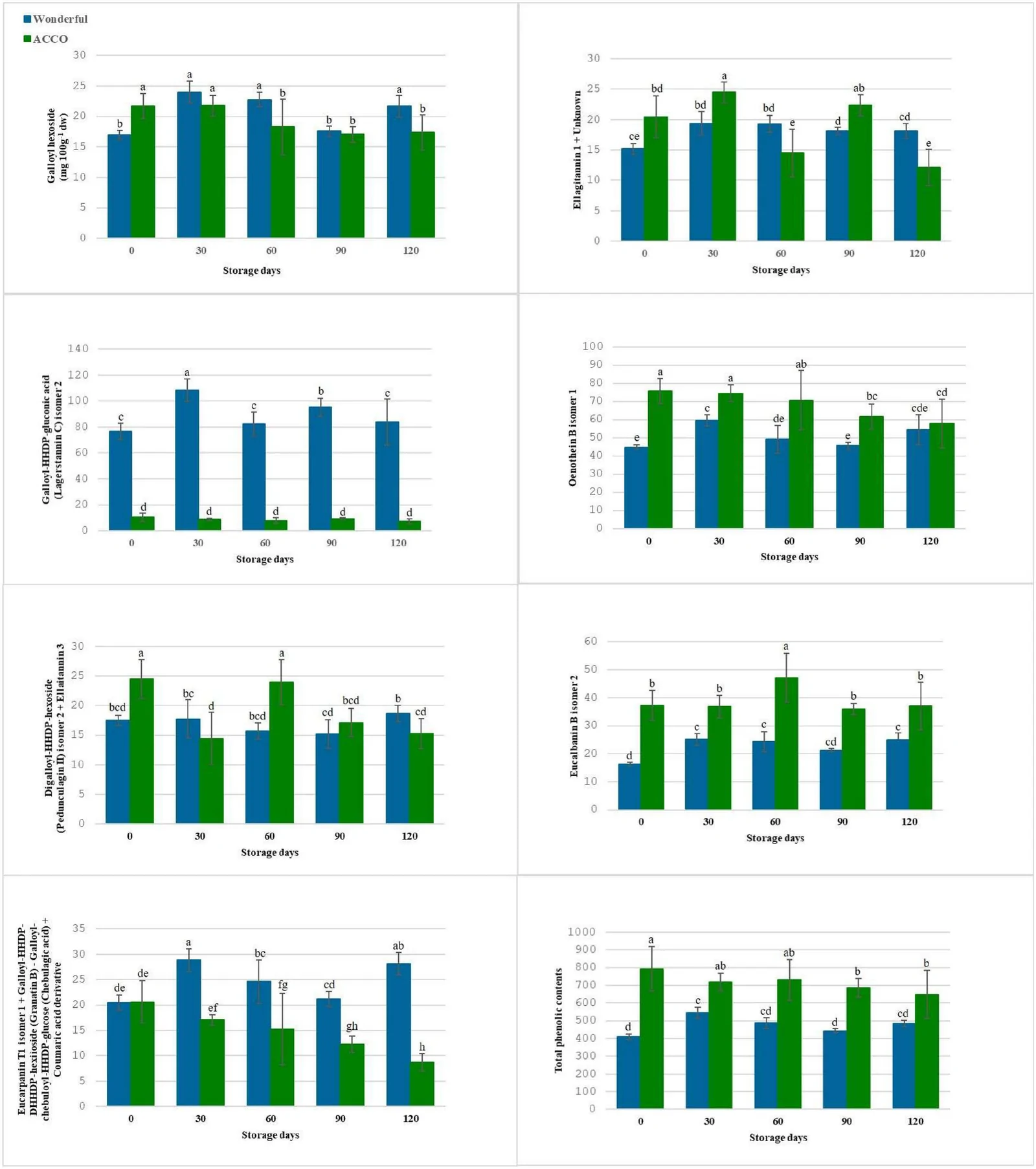
Effects of the interaction of cultivar and storage time (0, 30, 60, 90 and 120 days) on the concentration of the total phenolic compounds and of the most abundant phenolic compounds identified in dried Wonderful and Acco pomegranate samples. Each bar represents the mean and standard deviation (*n* = 3). Bar with same letters are not significantly different between cultivars at *p* ≤ 0.05 for a specific phenolic compound. The results of the analysis of all phenolic compounds are reported in the Table 1. dw, dry weight

**Table 2:** Effects of cultivar and storage time (0, 30, 60, 90 and 120 days) on the concentration of phenolic compounds (mg 100 g^−1^ dw) identified in dried Wonderful and Acco pomegranate samples. Values with different letters are significantly different among cultivars and storage time at *P* ≤ 0.05.

		**CULTIVAR**				
	**Phenolic Compounds**	**Wonderful**		**Acco**		
**14**	HHDP-gallagyl-hexoside (Punicalagin) isomer 1	nd		229.98		
**20**	Syringic acid-*O*-hexoside	2.25		nd		
**21**	HHDP-gallagyl-hexoside (Punicalagin) isomer 2	13.37	b	22.37	a	
**29**	Digalloyl-HHDP-hexoside (Pedunculagin II) isomer 1 + HHDP-gallagyl-hexoside (Punicalagin) isomer 3	40.04	b	67.17	a	
**38**	Ellagic acid hexoside + Syringic acid derivative	18.68	b	24.89	a	
**45**	Ellagic acid	19.09	a	7.71	b	
**46**	Eucarpanin T1 isomer 3	nd		19.24		

			**STORAGE DAYS**								
	***Phenolic Compounds***	***0d***		***30d***		***60d***		***90d***		***120d***	
**31**	Vanillic acid derivative 1	12.05	ab	13.96	a	13.97	a	12.32	ab	10.09	b
**45**	Ellagic acid	12.44	ab	14.82	a	14.36	a	11.65	b	13.74	ab

Abbreviations used: HHDP, hexahydroxydiphenoyl; nd, not detected.

Finally, the storage time affected the concentration of vanillic acid derivative 1 (Peak 31) and of ellagic acid (Peak 45) in the dry extracts of both cultivars (Table 2).

Fresh WF arils showed higher total phenolic content than fresh AC arils when expressed on a fresh-weight basis, whereas dried AC samples showed higher phenolic concentrations than dried WF samples when expressed on a dry-weight basis. Since the two datasets are expressed on different mass bases, these results do not allow direct conclusions regarding the effect of drying on phenolic retention.

This observation highlights differences between the studied WF and AC batches in the phenolic profiles observed after processing and during storage. Further studies are needed to determine whether these differences are reproducible and attributable to genotype effects.

### 3.3 Sensory characterization of dried pomegranate arils by descriptive analysis and TCATA

During the second trial, WF dried arils were selected and sensorially analysed through descriptive and dynamic tests.

The combination of descriptive analysis and TCATA demonstrates that both overall attribute intensity and temporal dynamics shift over time, reinforcing the value of integrating static and dynamic sensory approaches in shelf-life evaluation.

#### 3.3.1 Descriptive analysis

The descriptive analysis of dried arils across different storage periods revealed significant differences in most of the evaluated sensory attributes (Table 3). The sensory evolution of WF dried pomegranate arils during storage reflects the complex interplay between physicochemical degradation pathways and the preservation of specific quality attributes. Overall, the results indicated a progressive decline in most sensory attributes as storage time increased. However, several characteristics remained relatively stable up to 60 d (T60). Specifically, overall odor, along with pomegranate, red fruit, and raisin odor, showed no significant changes at this time point. A similar stability was observed for overall flavour, as well as pomegranate, floral, winey flavour notes, and bitterness. Regarding texture, moisture and crunchiness during mastication were the most stable attributes throughout the first 60 days of storage.

**Table 3:** Mean intensity scores of sensory attributes of dried arils of Wonderful cultivar at 0 (T0), 60 (T60), and 120 (T120) days of storage at 20 ± 1 °C. The number of assessors was ten (*n* = 10).

**Sensory attribute**	**T0**		**T60**		**T120**		***P-value***
Red Colour Intensity	6.7	a	6.2	b	5.2	c	***
Brilliance	3.5	a	3.2	b	2.4	c	***
Overall odor	2.7	a	2.4	a	1.8	b	***
Pomegranate odor	2.0	a	2.0	a	1.5	b	***
Red fruits odor	1.9	a	1.8	a	1.0	b	***
Flowers odor	1.6	a	1.2	b	1.0	b	***
Raisins odor	1.4	a	1.6	a	1.1	b	***
Winey odor	1.6		1.8		1.8		ns
Overall flavour	6.2	a	6.0	a	5.5	b	***
Pomegranate flavour	4.2	a	4.1	a	3.5	b	***
Red fruits flavour	4.3	a	4.0	b	3.6	c	***
Flowers flavour	2.6	a	2.4	a	1.8	b	***
Raisins flavour	3.2	a	2.8	b	2.7	b	***
Winey flavour	3.2	b	3.7	a	3.7	a	***
Acid	5.3		5.5		5.5		ns
Bitter	2.6	a	2.4	a	2.0	b	***
Sweet	3.3	a	2.9	b	2.7	b	***
Firmness	6.0		5.9		5.9		ns
Crunchiness at first bite	5.5	a	4.8	b	4.8	b	***
Crunchiness during mastication	5.3	a	5.2	a	4.8	b	**
Graininess	5.7	a	5.2	b	5.1	b	***
Mouth Coating	5.0		4.7		4.7		ns
Moisture	3.2	a	2.9	a	2.5	b	***
Astringency	4.5		4.8		4.8		ns
Tingling	5.1	a	4.6	b	4.4	b	***
Numbling	3.7	a	3.1	b	2.8	b	***

Different letters (a, b, c) indicate statistically significant differences between mean values according to one-way ANOVA followed by Tukey's post hoc test (ns: not significant *P* ≥ 0.05; **P < 0.01; ***P < 0.001).

Similar shelf-life patterns have been reported for dried fruits and dried pomegranate products, where aroma, flavour, and texture are generally preserved during the early stages of storage and progressively decline thereafter (Cardoso Reis et al., 2018; Dak et al., 2014; Sharma & Thakur, 2016). Therefore, our findings represent a sensory-based benchmark specifically for nitrogen-packaged dried pomegranate arils stored at room temperature and are consistent with previous work on fruits showing that although drying effectively extends shelf-life, the storage time induces changes in volatile composition and phenolic stability (Pu et al., 2018), which in turn affects the sensory perception. The stability observed in overall odor and specific aromatic notes (pomegranate, red fruits, raisins) up to 60 days aligns with findings that dried arils maintain much of their volatile and phenolic profile shortly following drying, with more pronounced degradation occurring only during the later stages of storage. For instance, Karaaslan et al. (2014) reported that key phenolic compounds in dried arils remain relatively unaffected immediately after drying, thereby preserving characteristic aroma notes. Prolonged storage generally leads to increases in fermentation-related volatiles such as ethanol and ethyl acetate, which correspond with declines in sensory quality and flavour acceptance in pomegranate products (Mayuoni-Kirshinbaum et al., 2013). Although the current study focuses on dried rather than minimally processed arils, the parallel phenomenon suggests that chemical transformations intensify as storage progresses beyond the early stages.

The relative stability of certain flavour attributes—such as pomegranate, floral, winey flavours, and bitterness—up to 60 days also mirrors the literature on dried pomegranate arils, where phenolic content and anthocyanins remain relatively stable in the early stages of storage. Adetoro et al. (2020) demonstrated that hot-air-dried arils retained significant levels of total phenolics and anthocyanins over a 12-week storage period, though antioxidant activity gradually declined. Since phenolic compounds contribute to bitterness and characteristic pomegranate flavour, their stability may explain the resilience of these sensory descriptors within the first two months.

Textural attributes also exhibited partial stability. Moisture retention and crunchiness remained consistent up to 60 days, which is consistent with observations that water activity and structural integrity of dried arils remain stable in the initial storage phase when packaged appropriately, with greater deterioration occurring only during extended storage periods (Sharma & Thakur, 2016).

However, the sensory stability observed up to 60 days should be considered a practical sensory shelf-life rather than a strict expiration limit. Nevertheless, similar trends have been reported for dried pomegranate products and other dehydrated fruits, where sensory deterioration becomes evident mainly after prolonged storage through losses of aroma intensity, flavour quality, and texture acceptability (Cardoso Reis et al., 2018; Dak et al., 2014). Studies on dried wild pomegranate arils packaged under different storage conditions have also reported progressive declines in sensory quality during storage, particularly for aroma-related attributes and texture parameters (Sharma & Thakur, 2016; Thakur et al., 2020).

Thus, the 60-day threshold identified in our study is consistent with previous observations and provides a useful reference for room-temperature storage of dried pomegranate snacks.

#### 3.3.2 Temporal check-all-that-apply

The TCATA approach was employed because it allows assessors to simultaneously describe multiple sensory perceptions over time, providing a detailed representation of the complex oral processing of dried fruits (Ares et al., 2015; Castura et al., 2016). In contrast, other temporal methods, such as Temporal Dominance of Sensations (TDS), focus only on the dominant sensation perceived at each moment. Although both methodologies have been successfully applied to characterize dynamic sensory perception, TCATA has been reported to provide richer information when multiple attributes coexist simultaneously throughout mastication (Ares et al., 2015). Since applications of dynamic sensory methodologies to dried pomegranate arils remain scarce, the present work expands current knowledge by describing temporal changes in flavour and texture perception during storage

TCATA Panel performance was assessed through the evaluation of assessor repeatability and replication. The results confirmed adequate panel consistency and reproducibility; therefore, the corresponding data are not reported here, as they fall outside the scope of the current work.

TCATA trajectories at different storage times (Fig.5) shared the same trend, the dynamic perception started for all samples with texture attribute (firmness and crunchiness) followed by (around 10 s) flavour attributes (flower, pomegranate, raisins and red fruits) and sweet taste, then acidity and graininess emerged, ending with tingling, astringency and numbing. With increasing storage duration (T120) there was a shift in the dynamic of the perception with acidity appearing earlier (time point 3–30 s) during the tasting phase as well as tingling (time point 4–40 s) compared to the other storage times (T0, T60) where the perception of those attributes were respectively at time point 4 and 5.

**Fig. 5:**
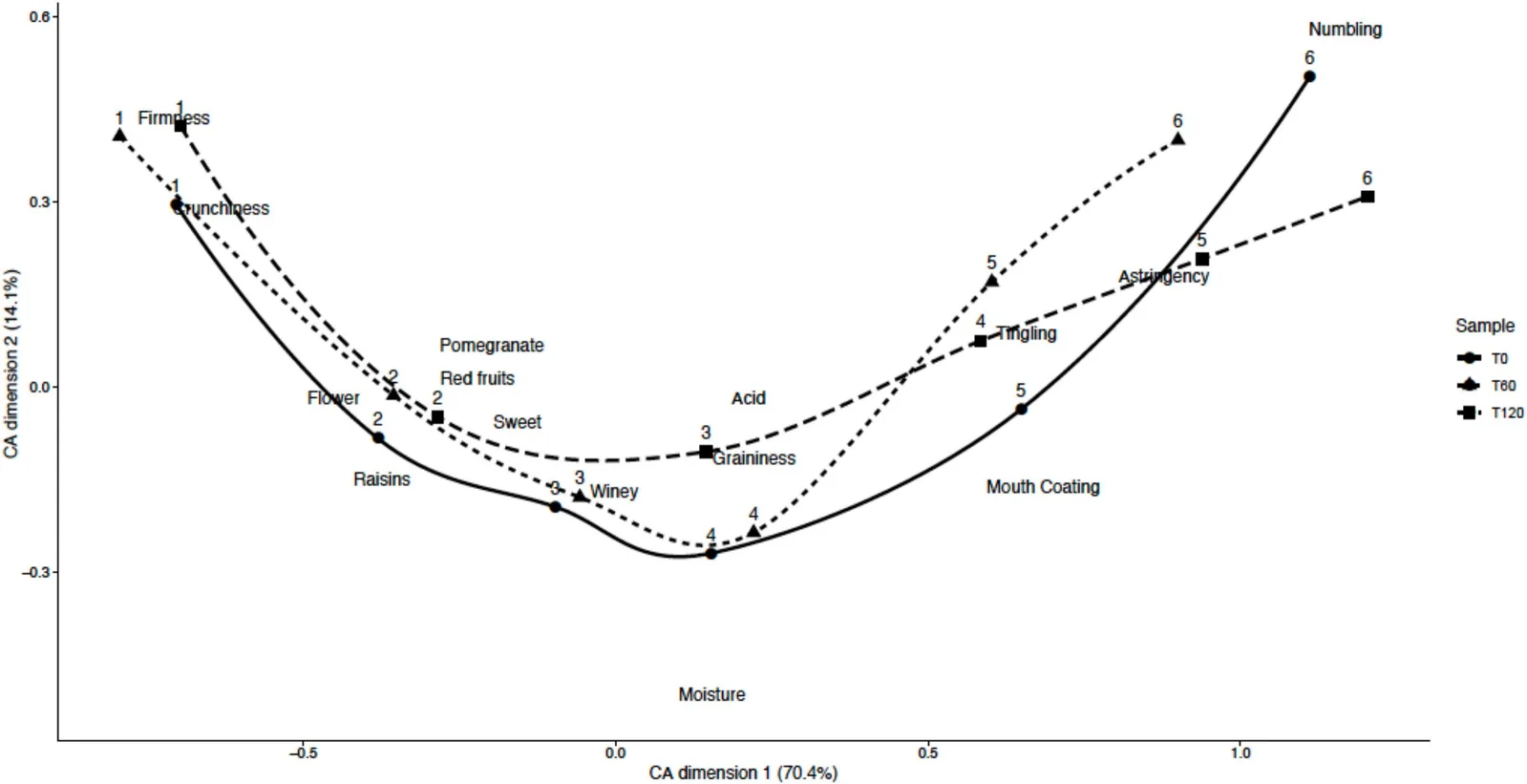
Correspondence analysis for dried arils at different storage times, showing Dimensions 1 and 2. Product trajectories are smoothed. Symbols and relative numbers (1–6) are shown at 10s increments.

These results suggested a modification of the temporal balance between taste, flavour and trigeminal sensations and is consistent with the descriptive analysis, which showed a progressive reduction in the intensity of characteristic aroma and flavour attributes during storage. As flavour-related sensations decreased in intensity, cross-modal masking effects may have been reduced, making acidic and trigeminal sensations more perceptually salient and therefore detectable earlier during oral processing (Pittari et al., 2020).

Indeed, TCATA approach is a relevant method to highlight sensory trajectories modification over time in products with different sensory profiles (Castura et al., 2016). The temporal shift observed at T120 may also be related to storage-induced modifications in phenolic composition since phenolic compounds contribute not only to flavour perception but also to astringent, tingling and mouthfeel sensations (Jayakalaiarasi et al., 2024). Although the present study did not include the determination of organic acids, volatile compounds, or anthocyanin degradation products, the observed evolution of the phenolic fraction suggests that compositional changes took place during storage and may have contributed to the sensory modifications detected. However, future studies are required to combine sensory evaluation with analysis of acids, volatile compounds and pigment degradation products and confirm a direct correlation of both sensory and chemical characterization. Overall, the TCATA results suggest that storage affects not only the intensity of sensory attributes, as shown by descriptive analysis, but also the temporal organization of sensory perception.

Additionally, the integration of biochemical and sensory results should be interpreted with caution because detailed sensory analyses (DA and TCATA) were performed on an independent batch of WF arils produced under the same processing conditions but not on the exact samples subjected to biochemical characterization. Therefore, the relationships discussed between phenolic composition and sensory perception represent qualitative associations based on common cultivar and storage trends rather than direct analytical correlations. Future studies should combine biochemical and sensory evaluations on the same experimental batches to better investigate the mechanistic relationships between phenolic compounds and sensory attributes.

## 4 Conclusions

This study evaluated the effects of cultivar, drying, and storage on the physicochemical, biochemical, and sensory properties of dried pomegranate arils from the Wonderful (WF) and Acco (AC) cultivars. Hot-air drying combined with nitrogen-flushed PP/PA packaging effectively maintained low water activity (aw <0.50) throughout storage and preserved product quality. However, microbiological analyses were not performed, and therefore further studies are required to confirm product safety and microbiological stability.

Within the experimental conditions adopted in this study, cultivar-associated differences were observed in several quality attributes; however, these findings were obtained from a single harvest and a single drying batch for each cultivar.

Although WF fresh arils showed higher total phenolic content than AC fresh arils when expressed on a fresh-weight basis, the dried AC samples exhibited higher phenolic concentrations than the dried WF samples when expressed on a dry-weight basis. Because fresh and dried samples were reported using different mass bases (fw and dw, respectively), direct conclusions regarding the effect of drying on the relative phenolic retention of the two cultivars cannot be drawn from the present data.

During storage, phenolic compounds decreased in both cultivars, but WF showed greater overall stability.

The dried batch obtained from WF exhibited greater colour retention and sensory stability up to 60 days, whereas the batch obtained from AC showed earlier deterioration of aroma- and taste-related attributes.

The combined results of descriptive sensory analysis and TCATA identified 60 days as the most appropriate sensory shelf-life under the tested conditions.

WF was identified as the most suitable cultivar within the context of this study and according to the parameters evaluated during the preliminary screening.

Under the conditions tested, the WF batch showed more favourable colour stability and sensory performance during storage than the AC batch. Nevertheless, because only one harvest and one drying batch were evaluated for each cultivar, these results should be regarded as preliminary indications and not as definitive evidence of cultivar superiority.

From an industrial perspective, these findings provide preliminary information on the behaviour of the studied batches and support the feasibility of hot-air drying combined with nitrogen-flushed packaging for producing shelf-stable pomegranate snacks. The identification of a 60-day sensory shelf-life may also support storage recommendations, distribution planning, and quality-based shelf-life labeling, only to Wonderful dried arils stored under the packaging and storage conditions adopted in this study.

Since each cultivar was represented by a single harvest and a single drying batch, the observed differences should be considered preliminary and require verification through independent harvests and replicated processing batches.

Finally, further studies should focus on microbiological validation and on packaging optimization to improve sensory retention during extended storage.

## CRediT authorship contribution statement

**Bernardo Pace:** Writing – review & editing, Writing – original draft, Conceptualization. **Chiara Medoro:** Validation, Investigation, Formal analysis, Data curation. **Michela Palumbo:** Writing – review & editing, Writing – original draft, Visualization, Methodology, Investigation, Data curation, Conceptualization. **Virginia Carbone:** Validation, Methodology, Investigation, Data curation. **Paola Minasi:** Investigation. **Marta Cianciabella:** Investigation, Data curation. **Stefano Predieri:** Writing – review & editing, Writing – original draft. **Maria Cefola:** Writing – review & editing, Writing – original draft, Visualization, Formal analysis, Conceptualization.

## Funding

This study was partially supported by CNR project NUTRAGE FOE-2021 DBA.AD005.225. Part of this study was conducted using facilities provided by PON R&I 2014–2020 PIR01_00017 CNRbiomics Project.

## Declaration of competing interest

The authors declare that they have no known competing financial interests or personal relationships that could have appeared to influence the work reported in this paper.

## Data Availability

The datasets supporting the findings of this study will be deposited in the Zenodo repository and will be publicly available upon publication.

## Appendix A Supplementary data

Supplementary material 1 Figure S1: Experimental flowchart. The acronym WF and AC are referred to Wonderful and Acco pomegranate cultivars, respectively; DA and TCATA stand for Descriptive Analysis and Temporal Check-All-That-Apply.

Supplementary material 2 Figure S2: Dried pomegranate arils of cultivars Wonderful (WF) and Acco (AC) after 0, 60, and 120 days of storage under modified atmosphere packaging (MAP) at 20 ± 1 °C in the dark.

Supplementary material 3 Figure S3: HPLC chromatograms of Wonderful pomegranate arils extracts: fresh, 520nm (A) and 280 nm (B); dry (time 0), 520nm (C) and 280 nm (D). For peak assignments, see Table S3.

Supplementary material 4 Table S1: Sensory attributes and descriptive five-point scale (5 = best quality, 1 = worst quality) used to evaluate the quality of dried aril of Wonderful and Acco cultivar.

Supplementary material 5 Table S2: Changes in quality parameters of fresh arils of Wonderful and Acco cultivar

Supplementary material 6 Table S3: List of phenolic compounds identified in extracts of fresh and dried Wonderful (WF) and Acco (AC) pomegranate samples. Specific quasi-molecular ions and fragment ions are reported for each compound.

Supplementary material 7 Table S4: Concentration of the total phenolic compounds and of individual phenolic compounds of fresh Wonderful and Acco pomegranate samples determined by HPLC-UV/Vis. Values with different letters are significantly different among cultivars at p ≤ 0.05. Data are mean of 3 replicates.

Supplementary material 8 Table S5: Effects of cultivar (Wonderful and Acco), storage time (0, 30, 60, 90, 120 days) and their interaction on colour parameters and water activity (a_w_) of dried arils of pomegranates.

Supplementary material 9 Table S6: Effects of cultivar (Wonderful and Acco), storage time (0, 30, 60, 90, 120 days) and their interaction on sensory attributes of dried arils of pomegranates.
