# Supplementary material for: Sensory and biochemical characterization of a snack based on dried pomegranate from two cultivars during shelf-life under modified atmosphere packaging
Source: Food Chem X. 2026 Nov 2;40:104742. doi: 10.1016/j.fochx.2026.104742 (PMC13769120; doi:10.1016/j.fochx.2026.104742)
Supplement: Supplementary file 1 [file mmc1.docx]

**Figure S1**. Experimental flowchart. The acronym WF and AC are referred to Wonderful and Acco pomegranate cultivars, respectively; DA and TCATA stand for Descriptive Analysis and Temporal Check-All-That-Apply.
