# Supplementary material for: Sensory and biochemical characterization of a snack based on dried pomegranate from two cultivars during shelf-life under modified atmosphere packaging
Source: Food Chem X. 2026 Nov 2;40:104742. doi: 10.1016/j.fochx.2026.104742 (PMC13769120; doi:10.1016/j.fochx.2026.104742)
Supplement: Supplementary file 2 [file mmc2.docx]

**Figure S2**. Dried pomegranate arils of cultivars Wonderful (WF) and Acco (AC) after 0, 60, and 120 days of storage under modified atmosphere packaging (MAP) at 20 ± 1 °C in the dark.
