# Supplementary material for: Sensory and biochemical characterization of a snack based on dried pomegranate from two cultivars during shelf-life under modified atmosphere packaging
Source: Food Chem X. 2026 Nov 2;40:104742. doi: 10.1016/j.fochx.2026.104742 (PMC13769120; doi:10.1016/j.fochx.2026.104742)
Supplement: Supplementary file 3 [file mmc3.docx]

**Figure S3.** HPLC chromatograms of Wonderful pomegranate arils extracts: fresh, 520nm (A) and 280 nm (B); dry (time 0), 520nm (C) and 280 nm (D). For peak assignments, see Table S3.
