# Supplementary material for: Sensory and biochemical characterization of a snack based on dried pomegranate from two cultivars during shelf-life under modified atmosphere packaging
Source: Food Chem X. 2026 Nov 2;40:104742. doi: 10.1016/j.fochx.2026.104742 (PMC13769120; doi:10.1016/j.fochx.2026.104742)
Supplement: Supplementary file 4 [file mmc4.docx]

**Table S1.** Sensory attributes and descriptive five-point scale (5 = best quality, 1 = worst quality) used to evaluate the quality of dried aril of Wonderful and Acco cultivar.

| **Attribute** | **5** | **4** | **3** | **2** | **1** |
| --- | --- | --- | --- | --- | --- |
| **Visual Appearance** | Bright, uniform red/orange, intact arils, plump | Mostly uniform, minor colour variations, slightly shriveled | Some uneven colour or slightly broken | Many broken or shriveled arils, dull | Very dull, heavily shriveled, fragmented |
|  |  |  |  |  |  |
| **Characteristics smell** | Sweet, fruity, pleasant, natural dried aroma | Mostly pleasant, slight mustiness | Mild aroma, slightly off | Noticeable off odors, slightly fermented | Strongly unpleasant, chemical, or musty |
|  |  |  |  |  |  |
| **Dislike Smell** | No unpleasant smell | Slight off odor | Noticeable unpleasant odor | Strong off odor | Very strong, extremely unpleasant |
|  |  |  |  |  |  |
| **Taste** | Sweet-tart, rich, full flavor, natural | Mostly sweet-tart, minor loss of flavor | Noticeably less flavorful, slightly bitter or sour | Bland, overly sour, or slightly off | Extremely bland, bitter, or off-putting |
|  |  |  |  |  |  |
| **Crunchiness** | Slightly crisp but chewy, pleasant bite | Mostly crisp, slightly tough | Moderate crunchiness, some hard bits | Hard, tough, difficult to bite | Very hard, unpleasantly tough |
|  |  |  |  |  |  |
| **Chewiness** | Low-medium chewiness, easy to eat | Slightly chewy, still pleasant | Moderate chewiness, noticeable effort | Very chewy, somewhat unpleasant | Extremely tough, difficult to chew |
|  |  |  |  |  |  |
| **Overall Quality** | Excellent, balanced flavor and texture, very appealing | Good, minor issues | Average, acceptable | Poor, several negative traits | Very poor, inedible or unacceptable |
