# Supplementary material for: Sensory and biochemical characterization of a snack based on dried pomegranate from two cultivars during shelf-life under modified atmosphere packaging
Source: Food Chem X. 2026 Nov 2;40:104742. doi: 10.1016/j.fochx.2026.104742 (PMC13769120; doi:10.1016/j.fochx.2026.104742)
Supplement: Supplementary file 5 [file mmc5.docx]

**Table S2.** Changes in quality parameters of fresh arils of Wonderful and Acco cultivar

| **Parameters** | **Wonderful** | **Acco** | ***P*-value** |
| --- | --- | --- | --- |
| pH | 3.27 | 2.90 | ns |
| TSS | 16.07 a | 14.43 b | ** |
| TA (%) | 3.33 a | 2.49 b | ** |
| TP (mg gallic acid 100 g^-1^ fw) | 259.33 a | 196.43 b | * |
| AA (mg Trolox 100 g^-1^ fw) | 304.71 a | 240.12 b | ** |
| Results are given as mean values of 6 samples (3 replicates x 2 cultivar). Different letters within the same row indicate significant differences among pomegranate cultivars according to the LSD post hoc test (*p* ≤ 0.05). The level of statistical significance is reported as follows: ns = not significant; * = *p* ≤ 0.05; ** = *p* ≤ 0.01. | | | |
