# Supplementary material for: Sensory and biochemical characterization of a snack based on dried pomegranate from two cultivars during shelf-life under modified atmosphere packaging
Source: Food Chem X. 2026 Nov 2;40:104742. doi: 10.1016/j.fochx.2026.104742 (PMC13769120; doi:10.1016/j.fochx.2026.104742)
Supplement: Supplementary file 6 [file mmc6.docx]

**Table S3.** List of phenolic compounds identified in extracts of fresh and dried Wonderful (WF) and Acco (AC) pomegranate samples. Specific quasi-molecular ions and fragment ions are reported for each compound.

| **Peak** | **tR *(min)*** | **MS**  ***m/z*** | **MS^n^ ions**  ***m/z*** | **Tentative Assignment** | **WF**  **fresh** | **WF**  **dry** | **AC**  **fresh** | **ACdry** |
| --- | --- | --- | --- | --- | --- | --- | --- | --- |
| **1** | 10.89 | 627 [M]^+^ | MS^2^ [627]: 465, 303  MS^3^ [303]: 285, 275, 257, 229, 201, 179, 153 | Delphinidin 3,5-diglucoside | x | x | x | x |
| **2** | 13.89 | 611 [M]^+^ | MS^2^ [611]: 449, 287  MS^3^ [287]: 269, 241, 213, 189, 175, 161, 149, 137 | Cyanidin 3,5-diglucoside | x | x | x | x |
| **3** | 16.87 | 465 [M]^+^ | MS^2^ [465]: 303  MS^3^ [303]: 285, 257, 229, 201, 173, 165 | Delphinidin 3-glucoside | x | x | x | x |
| **4** | 20.07 | 449 [M]^+^ | MS^2^ [449]:287  MS^3^ [287]: 269, 241, 213, 189, 185, 145, 137 | Cyanidin 3-glucoside | x | x | x | x |
| **5** | 23.28 | 433 [M]^+^ | MS^2^ [433]:271  MS^3^ [271]: 253, 225, 197, 173, 169, 145, 121 | Pelargonidin 3-glucoside | x | x | x |  |
| **6** | 26.25 | 419 [M]^+^ | MS^2^ [419]: 287  MS^3^ [287]: 269, 241, 213, 189, 175, 149, 137 | Cyanidin 3-pentoside | x | x | x |  |
| **7** | 27.08 | 419 [M]^+^ | MS^2^ [419]: 287  MS^3^ [287]: 269, 241, 213, 189, 175, 149, 137 | Cyanidin 3-pentoside | x | x | x |  |
| **Peak** |  | **MS**  ***m/z*** | **MS^n^ ions**  ***m/z*** | **Tentative Assignment** | **WF**  **fresh** | **WF**  **dry** | **AC**  **fresh** | **ACdry** |
| **8** | 2.36 | 353 [M-H]^-^  391 [M-H]^-^ | MS^2^ [353]: 293, 191, 173, 155, 111  MS^3^ [173]: 111  MS^4^ [111]: 67  MS^2^ [391]: 373, 217, 191  MS^3^ [217]: 155, 111  MS^4^ [111]: 67 | Caffeoyl-isocitric acid  Citric acid derivative | x | x |  | x |
| **9** | 2.56 | 191 [M-H]^-^ | MS^2^ [191]: 173, 111  MS^3^ [173]: 129, 111, 173  MS^4^ [111]: 67 | Citric acid | x | x | x | x |
| **10** | 2.86 | 331 [M-H]^-^ | MS^2^ [331]: 271, 211, 193, 169, 125  MS^3^ [169]: 125  MS^3^ [271]: 253, 211, 193, 169 | Galloyl-hexoside | x | x | x | x |
| **11** | 3.55 | 645 [M-H]^-^  649 [M-H]^-^  633 [M-H]^-^ | MS^2^ [645]: 483, 357, 303  MS^3^ [483]: 439, 357, 303, 237, 177  MS^4^ [303]: 275, 207, 177, 151  MS^2^ [649]: 631, 497, 301  MS^3^ [497]: 301  MS^4^ [301]: 257, 229, 185, 145  MS^2^ [633]: 481, 463, 301, 275, 257  MS^3^ [301]: 284, 257, 229, 185 | Unknown  Galloyl-HHDP-gluconic acid  (Lagerstannin C) isomer 1  Galloyl-HHDP-hexoside | x | x | x | x |
| **12** | 3.77 | 643 [M-H]^-^  645 [M-H]^-^ | MS^2^ [643]: 625, 505, 481, 463, 355, 301, 283  MS^3^ [463]: 301, 283  MS^4^ [283]: 255, 239, 227  MS^2^ [645]: 483, 357, 303  MS^3^ [483]: 439, 357, 303, 237, 177  MS^4^ [303]: 275, 207, 177, 151 | Ellagitannin 1  Unknown | x | x | x | x |
| **13** | 4.05 | 527 [M-H]^-^ | MS^2^ [527]: 509, 353, 329  MS^3^ [353]: 173, 111  MS^4^ [173]: 111 | Unknown |  | x |  | x |
| **14** | 6.02 | 1083 [M-H]^-^ | MS^2^ [1083]:1065, 781, 721, 601, 575, 549  MS^3^ [781]: 721, 665, 601, 575, 299  MS^4^ [601]: 555, 299, 271 | HHDP-gallagyl-hexoside (Punicalagin) isomer 1 |  |  |  | x |
| **15** | 6.32 | 649 [M-H]^-^ | MS^2^ [649]: 497, 301  MS^3^ [497]: 301  MS^4^ [301]: 257, 229, 185 | Galloyl-HHDP-gluconic acid  (Lagerstannin C) isomer 2 | x | x | x | x |
| **16** | 6.86 | 368 [M-H]^-^  629 [M-H]^-^ | MS^2^ [368]: 324, 188, 179, 161, 144, 131, 119, 113  MS^3^ [179]: 161, 149, 143, 131, 119, 113  MS^2^ [629]: 467, 449, 341, 287  MS^3^ [467]: 423, 357, 341, 287  MS^4^ [287]: 259, 243, 219, 215, 203, 177, 175 | Unknown  Unknown | x | x | x | x |
| **17** | 8.30 | 643 [M-H]^-^ | MS^2^ [643]: 625, 481, 463, 355, 301, 283  MS^3^ [481]: 355, 329, 319, 301, 283, 257, 193, 175  MS^4^ [301]: 283, 273, 259, 257, 233, 175 | Ellagitannin 2 | x | x | x | x |
| **18** | 9.11 | 643 [M-H]^-^ | MS^2^ [643]: 625, 481, 463, 355, 301, 283  MS^3^ [481]: 355, 329, 319, 301, 283, 257, 193, 175  MS^4^ [301]: 283, 273, 259, 257, 233, 175 | Ellagitannin 2 isomer | x |  | x |  |
| **19** | 7.96 | 341 [M-H]^-^ | MS^2^ [341]: 179  MS^3^ [179]: 135 | Caffeic acid hexoside |  | x |  | x |
| **20** | 8.24 | 359 [M-H]^-^ | MS^2^ [359]:197  MS^3^ [197]: 182, 153 | Syringic acid-O-hexoside |  | x |  |  |
| **21** | 9.93 | 1083 [M-H]^-^ | MS^2^ [1083]:1065, 781, 721, 601, 575, 549  MS^3^ [781]: 721, 665, 601, 575, 299  MS^4^ [601]: 555, 299, 271 | HHDP-gallagyl-hexoside (Punicalagin) isomer 2 | x | x | x | x |
| **22** | 10.20 | 329 [M-H]^-^ | MS^2^ [329]:311, 269, 239, 209, 167  MS^3^ [167]: 152, 123, 108 | Vanillic acid glucoside | x | x |  |  |
| **23** | 10.84 | 341 [M-H]^-^ | MS^2^ [341]: 179  MS^3^ [179]: 135 | Caffeic acid hexoside |  | x | x | x |
| **24** | 11.48 | 479 [M-H]^-^ | MS^2^ [479]: 461, 435, 317, 299, 191  MS^3^ [299]: 281, 271, 255, 243  MS^4^ [243]: 225, 215, 199 | Unknown | x |  | x |  |
| **25** | 11.87 | 951 [M-H]^-^ | MS^2^ [951]: 907, 783  MS^3^ [907]: 783, 745, 605, 481, 301, 275  MS^4^ [783]: 481, 301, 275 | HHDP-valoneoyl-glucoside | x | x | x | x |
| **26** | 12.70 | 1417 [M-H]^-^  708 [M-2H]^2-^ | MS^2^ [1417]: 1247, 1115, 1085, 1059, 785, 783, 765, 633, 613, 451, 301  MS^3^ [633]: 615, 481, 463, 301, 275, 249  MS^3^ [765]: 747, 721, 613, 597, 427, 301, 275  MS^3^ [785]: 767, 633, 483, 419, 301, 275,249  MS^2^ [708]: 1247, 1085, 783, 765, 633, 613, 301  MS^3^ [633]: 615, 481, 301, 275, 249  MS^4^ [301]: 257, 229, 185 | Ellagitannin (Camptothin A) | x | x | x | x |
| **27** | 13.23 | 481 [M-H]^-^ | MS^2^ [481]: 319, 301 | HHDP- hexoside | x | x | x |  |
| **28** | 13.80 | 1567 [M-H]^-^  783 [M-2H]^2-^ | MS^2^ [1567]: 1549, 1397, 1209, 935, 915, 783, 765, 753  MS^3^ [765]: 747, 613, 597, 533, 427, 401, 301, 275  MS^3^ [935]: 917, 783, 765, 451, 427, 401, 301, 275  MS^4^ [765]: 747, 703, 597, 533, 427, 401, 399, 301, 275  MS^2^ [783]: 1379, 935, 769, 765, 613, 451, 301, 275, 249 | Oenothein B isomer 1 | x | x | x | x |
| **29** | 14.93 | 785 [M-H]^-^  1083 [M-H]^-^ | MS^2^ [785]: 767, 633, 615, 483, 463, 419, 331, 301, 275, 249  MS^3^ [633]: 615, 463, 421, 301, 275, 249  MS^3^ [483]: 331, 313, 295, 169  MS^3^ [301]: 257, 229, 185  MS^2^ [1083]: 781, 721, 601, 575  MS^3^ [781]: 721,  601, 575, 299  MS^4^ [601]: 299, 271 | Digalloyl-HHDP-hexoside (Pedunculagin II) isomer 1  HHDP-gallagyl-hexoside (Punicalagin) isomer 3 | x | x | x | x |
| **30** | 15.35 | 325 [M-H]^-^ | MS^2^ [325]: 307, 187, 163, 145, 119  MS^3^ [163]: 119 | Coumaric acid hexoside |  | x | x |  |
| **31** | 16.60 | 525 [M-H]^-^ | MS^2^ [525]: 363, 315, 167  MS^3^ [363]: 315, 195, 167, 151  MS^4^ [167]: 151, 123 | Vanillic acid derivative 1 | x | x | x | x |
| **32** | 17.52 | 525 [M-H]^-^ | MS^2^ [525]: 363, 315, 195, 167  MS^3^ [363]: 315, 195, 167, 151 | Vanillic acid derivative 2 | x | x | x | x |
| **33** | 17.46 | 355 [M-H]^-^ | MS^2^ [355]: 337, 311, 193, 175  MS^3^ [193]: 178, 145, 134 | Ferulic acid hexoside |  | x |  |  |
| **34** | 18.02 | 387 [M-H]^-^ | MS^2^ [387]: 369, 343, 309, 231, 207, 163  MS^3^ [207]: 192, 175, 163  MS^3^ [309]: 289, 231 | Medioresinol |  | x |  |  |
| **35** | 19.30 | 1085 [M-H]^-^  1567 [M-H]^-^  783 [M-2H]^2-^ | MS^2^ [1085]: 783, 765, 633, 597  MS^3^ [765]: 613, 597, 595, 301, 275  MS^2^ [1567]: 1549, 1209, 935, 915, 783, 765  MS^3^ [765]: 613, 597, 533, 401, 301  MS^2^ [783]: 1265, 935, 933, 1379, 1265, 935, 933, 769, 765, 633, 481, 301  MS^3^ [765]: 747, 613, 597, 533, 463, 427, 399, 301, 275 | Tri-HHDP-hexoside  Oenothein B isomer 2 | x |  | x | x |
| **36** | 20.12 | 1085 [M-H]^-^  1567 [M-H]^-^  783 [M-2H]^2-^ | MS^2^ [1085]: 783, 765, 633, 597  MS^3^ [765]: 613, 597, 595, 301, 275  MS^2^ [1567]: 1549, 1209, 935, 915, 783, 765  MS^3^ [765]: 613, 597, 533, 401, 301  MS^2^ [783]: 1265, 933, 765, 1265, 935, 633, 613, 481, 301 | Tri-HHDP-hexoside  Oenothein B isomer 3 | x | x | x | x |
| **37** | 20.65 | 785 [M-H]^-^  643 [M-H]^-^ | MS^2^ [785]: 767, 633, 615, 483, 463, 419, 331, 301, 275, 249  MS^3^ [633]: 615, 463, 421, 301, 275, 249  MS^3^ [483]: 331, 313, 295, 169  MS^3^ [301]: 257, 229, 185  MS^2^ [643]: 625, 463, 481, 345  MS^3^ [481]: 463, 345, 315, 255, 165, 153  MS^4^ [345]: 327, 183, 165, 139  MS^4^ [315]: 255, 195, 177, 153 | Digalloyl-HHDP-hexoside (Pedunculagin II) isomer 2  Ellagitannin 3 | x | x | x | x |
| **38** | 23.04 | 463 [M-H]^-^  555 [M-H]^-^ | MS^2^ [463]: 301  MS^3^ [301]: 257, 229, 185, 145  MS^3^ [301]: 257, 229, 185, 145  MS^2^ [555]: 537, 393, 197  MS^3^ [393]: 375, 345, 197, 195, 182, 165, 151  MS^4^ [197]: 182, 153 | Ellagic acid hexoside  Syringic acid derivative | x | x | x | x |
| **39** | 23.53 | 551 [M-H]^-^ | MS^2^ [551]: 389, 341, 193  MS^3^ [389]: 341, 193, 165  MS^4^ [193]: 178, 149, 134  MS^4^ [341]: 326, 309, 297, 282, 265, 250, 235, 233, 205 | Ferulic acid-C-hexoside  derivative | x | x | x |  |
| **40** | 25.48 | 785 [M-H]^-^ | MS^2^ [785]: 767, 633, 615, 483, 463, 419, 331, 301, 275, 249  MS^3^ [615]: 571, 445, 419, 301, 275  MS^3^ [419]: 401, 359, 329, 293, 275, 249, 219  MS^3^ [301]: 257, 229, 185 | Digalloyl-HHDP-hexoside (Pedunculagin II) isomer 3 | x |  | x |  |
| **41** | 26.18 | 1569 [M-H]^-^  784 [M-2H]^2-^ | MS^2^ [1569]: 1550, 1531, 1399, 1247, 1085, 935, 785, 765, 597, 451  MS^3^ [765]: 747, 721, 613, 597, 533, 427, 401, 301, 275  MS^4^ [597]: 579, 553, 509, 445, 427, 401, 355, 301, 275, 249  MS^3^ [935]: 917, 891, 811, 783, 765, 613, 451, 425, 327  MS^4^ [451]: 433, 405, 395, 377, 327, 301  MS^3^ [785]: 767, 757, 633, 615, 533, 483, 419, 301, 275, 249  MS^2^ [784]: 1085, 935, 785, 765, 699, 633, 623, 529, 483, 419, 301, 275, 249  MS^3^ [765]: 915, 766, 721, 613, 597, 553, 533, 445, 427, 301, 275  MS^4^ [597]: 553, 535, 509, 445, 427, 401, 383, 325, 301, 275  MS^3^ [935]: 936, 917, 811, 783, 765, 755, 739, 696, 613, 487, 483, 451, 425, 367, 301 | Eucalbanin B isomer 1 | x | x | x | x |
| **42** | 27.03 | 1569 [M-H]^-^  784 [M-2H]^2-^ | MS^2^ [1569]: 1550, 1531, 1399, 1247, 1085, 935, 785, 765, 597, 451  MS^3^ [765]: 747, 721, 613, 597, 533, 427, 401, 301, 275  MS^4^ [597]: 579, 553, 509, 445, 427, 401, 355, 301, 275, 249  MS^3^ [935]: 917, 891, 811, 783, 765, 613, 451, 425, 327  MS^4^ [451]: 433, 405, 395, 377, 327, 301  MS^3^ [785]: 767, 757, 633, 615, 533, 483, 419, 301, 275, 249  MS^2^ [784]: 1085, 935, 785, 765, 699, 633, 623, 529, 483, 419, 301, 275, 249  MS^3^ [765]: 915, 766, 721, 613, 597, 553, 533, 445, 427, 301, 275  MS^4^ [597]: 553, 535, 509, 445, 427, 401, 383, 325, 301, 275  MS^3^ [935]: 936, 917, 811, 783, 765, 755, 739, 696, 613, 487, 483, 451, 425, 367, 301 | Eucalbanin B isomer 2 | x | x | x | x |
| **43** | 29.78 | 1176 [M-2H]^2-^  784 [M-3H]^3-^  951 [M-H]^-^  953 [M-H]^-^  429 [M-H]^-^ | MS^2^ [1176]: 1869, 1719, 1569, 1549, 1417, 1399, 1247, 1091, 935, 785, 765, 633, 615, 533, 483, 463, 451, 419  MS^3^ [1569]: 1913, 1398, 1379, 1245, 1076, 935, 786, 598, 451  MS^2^ [784]: 1399, 1267, 1085, 935, 765, 633, 615, 529, 483, 301, 275  MS^3^ [765]: 916, 766, 721, 613, 597, 553, 533, 445, 427, 401, 301, 275  MS^4^ [597]: 553, 509, 445, 427, 399, 301, 275  MS^3^ [301]: 257, 299, 185  MS^2^ [951]: 933, 915, 765, 613, 445, 301  MS^3^ [933]: 915,  897, 765, 631, 613, 463, 445, 301, 275  MS^4^ [301]: 257, 229  MS^2^ [953]: 935, 917, 909, 891, 853, 785, 633, 463, 343, 301  MS^3^ [935]: 917, 899, 765, 633, 615, 463, 445, 343, 301, 275  MS^4^ [301]: 257, 229  MS^2^ [429]: 325, 307, 265, 235, 163  MS^3^ [163]: 145, 127, 119, 103, 89 | Eucarpanin T1 isomer 1  Galloyl-HHDP-DHHDP-hexoside (Granatin B)  Galloyl-chebuloyl-HHDP-glucose (Chebulagic acid)  Coumaric acid derivative | x | x | x | x |
| **44** | 31.21 | 1176 [M-2H]^2-^  784 [M-3H]^3-^  447 [M-H]^-^  433[M-H]^-^ | MS^2^ [1176]: 1869, 1719, 1569, 1549, 1417, 1399, 1247, 1091, 935, 785, 765, 633, 615, 533, 483, 463, 451, 419  MS^2^ [784]: 1399, 1267, 1085, 935, 765, 699, 633, 615, 543, 483, 301  MS^3^ [765]: 766, 721, 613, 597, 553, 533, 443, 427, 401,301, 275  MS^2^ [447]: 300, 301  MS^3^ [301]: 257, 229  MS^2^ [433]: 301  MS^3^ [301]: 257, 229, 185 | Eucarpanin T1 isomer 2  Ellagic acid-deoxyhexoside  Ellagic acid-pentoside | x | x | x | x |
| **45** | 32.28 | 301 M-H]^-^ | MS^2^ [301]: 257, 229, 185 | Ellagic acid | x | x | x | x |
| **46** | 32.69 | 1176 [M-2H]^2-^  784 [M-3H]^3-^ | MS^2^ [1176]: 1869, 1719, 1569, 1549, 1417, 1399, 1247, 1091, 935, 785, 765, 633, 615, 533, 483, 463, 451  MS^2^ [784]: 1399, 1267, 1085, 935, 765, 699, 633, 615, 543, 483, 301  MS^3^ [765]: 766, 721, 613, 597, 553, 533, 443, 427, 401, 301, 275 | Eucarpanin T1 isomer 3 | x |  | x | x |
| **47** | 33.31 | 507 [M-H]^-^ | MS^2^ [507]: 489, 345, 327, 315, 209  MS^3^ [327]: 312, 296, 283  MS^3^ [345]: 327, 315, 301, 221, 209 | Pomegralignan | x | x | x | x |
| **48** | 35.79 | 937 [M-H]^-^ | MS^2^ [937]: 919, 893, 785, 767, 635, 617, 597, 483, 465, 445, 419, 313, 301, 275, 257 | Trigalloyl-HHDP-hexoside | x |  | x |  |
| **49** | 38.30 | 533 [M-H]^-^ | MS^2^ [533]: 515, 503,473, 425, 395, 371, 353, 341  MS^3^ [353]: 338 | Guaiacyl(8-5)ferulic acid hexoside | x | x | x |  |

Abbreviations used: HHDP, hexahydroxydiphenoyl; DHHDP, dehydrohexahydroxydiphenoyl: x, detected
