# Supplementary material for: Sensory and biochemical characterization of a snack based on dried pomegranate from two cultivars during shelf-life under modified atmosphere packaging
Source: Food Chem X. 2026 Nov 2;40:104742. doi: 10.1016/j.fochx.2026.104742 (PMC13769120; doi:10.1016/j.fochx.2026.104742)
Supplement: Supplementary file 7 [file mmc7.docx]

**Table S4.** Concentration of the total phenolic compounds and of individual phenolic compounds of fresh Wonderful and Acco pomegranate samples determined by HPLC-UV/Vis. Values with different letters are significantly different among cultivars at p ≤ 0.05. Data are mean of 3 replicates.

|  | ***Phenolic Compounds*** | ***Wonderful***  ***(mg 100g****^-1^* ***fw)*** | |  | | ***Acco***  ***(mg 100g****^-1^* ***fw)*** | |  | |
| --- | --- | --- | --- | --- | --- | --- | --- | --- | --- |
| **1** | Delphinidin 3,5-diglucoside | 7.17 | a | | 4.33 | | b | |  |
| **2** | Cyanidin 3,5-diglucoside | 4.31 | a | | 2.35 | | b | |  |
| **3** | Delphinidin 3-glucoside | 8.22 | a | | 2.58 | | b | |  |
| **4** | Cyanidin 3-glucoside | 5.18 | a | | 1.53 | | b | |  |
| **5** | Pelargonidin 3-glucoside | 0.38 | a | | 0.09 | | b | |  |
| **6** | Cyanidin 3-pentoside | 0.23 | a | | 0.08 | | b | |  |
| **7** | Cyanidin 3-pentoside | 0.08 | a | | 0.04 | | b | |  |
| **10** | Galloyl-hexoside | 6.63 | a | | 4.59 | | b | |  |
| **11** | Galloyl-HHDP-gluconic acid  (Lagerstannin C) isomer 1 + Galloyl-HHDP-hexoside + Unknown | 2.30 | ns | | 2.86 | | ns | |  |
| **12** | Ellagitannin 1 + Unknown | 2.86 | a | | 1.98 | | b | |  |
| **15** | Galloyl-HHDP-gluconic acid  (Lagerstannin C) isomer 2 | 2.92 | ns | | 2.65 | | ns | |  |
| **17** | Ellagitannin 2 | 1.74 | a | | 1.56 | | b | |  |
| **18** | Ellagitannin 2 isomer | 2.07 | a | | 1.35 | | b | |  |
| **21** | HHDP-gallagyl-hexoside (Punicalagin) isomer 2 | 4.77 | a | | 2.28 | | b | |  |
| **22** | Vanillic acid glucoside | 2.39 |  | | nd | |  | |  |
| **23** | Caffeic acid hexoside | nd |  | | 1.60 | |  | |  |
| **25** | HHDP-valoneoyl-glucoside | 7.69 | a | | 9.60 | | b | |  |
| **26** | Ellagitannin (Camptothin A) | 4.10 | a | | 3.33 | | b | |  |
| **27** | HHDP- hexoside | 1.53 | ns | | 1.62 | | ns | |  |
| **28** | Oenothein B isomer 1 | 30.09 | a | | 24.84 | | b | |  |
| **29** | Digalloyl-HHDP-hexoside (Pedunculagin II) isomer 1 + HHDP-gallagyl-hexoside (Punicalagin) isomer 3 | 17.89 | a | | 11.17 | | b | |  |
| **30** | Coumaric acid hexoside | nd |  | | 1.28 | |  | |  |
| **31** | Vanillic acid derivative 1 | 11.81 | a | | 5.84 | | b | |  |
| **32** | Vanillic acid derivative 2 | 6.31 | a | | 3.95 | | b | |  |
| **35** | Tri-HHDP-hexoside + Oenothein B isomer 2 | 4.43 | a | | 3.24 | | b | |  |
| **36** | Tri-HHDP-hexoside + Oenothein B isomer 3 | 2.70 | ns | | 2.43 | | ns | |  |
| **37** | Digalloyl-HHDP-hexoside (Pedunculagin II) isomer 2 + Ellagitannin 3 | 17.59 | a | | 9.67 | | b | |  |
| **38** | Ellagic acid hexoside + Syringic acid derivative | 4.41 | ns | | 4.57 | | ns | |  |
| **39** | Ferulic acid-C-hexoside  derivative | 2.75 | a | | 1.96 | | b | |  |
| **40** | Digalloyl-HHDP-hexoside (Pedunculagin II) isomer 3 | 2,00 | ns | | 1.49 | | ns | |  |
| **41** | Eucalbanin B isomer 1 | 8.76 | ns | | 7.71 | | ns | |  |
| **42** | Eucalbanin B isomer 2 | 16.51 | ns | | 17.67 | | ns | |  |
| **43** | Eucarpanin T1 isomer 1 + Galloyl-HHDP-DHHDP-hexoside (Granatin B) + Galloyl-chebuloyl-HHDP-glucose (Chebulagic acid) + Coumaric acid derivative | 17.08 | ns | | 17.19 | | ns | |  |
| **44** | Eucarpanin T1 isomer 2 + Ellagic acid-deoxyhexoside + Ellagic acid-pentoside | 9.19 | b | | 10.11 | | a | |  |
| **45** | Ellagic acid | 4.20 | a | | 2.13 | | b | |  |
| **46** | Eucarpanin T1 isomer 3 | 2.87 | ns | | 2.97 | | ns | |  |
| **48** | Trigalloyl-HHDP-hexoside | 2.46 | a | | 1.60 | | b | |  |
| **49** | Guaiacyl(8-5)ferulic acid hexoside | 0.99 | a | | 0.79 | | b | |  |
|  | Total Phenolic Contents | 226.62 | a | | 175.10 | | b | |  |

Abbreviations used: HHDP, hexahydroxydiphenoyl; DHHDP, dehydrohexahydroxydiphenoyl; ns, not significant; nd, not detected.
