# Supplementary material for: Sensory and biochemical characterization of a snack based on dried pomegranate from two cultivars during shelf-life under modified atmosphere packaging
Source: Food Chem X. 2026 Nov 2;40:104742. doi: 10.1016/j.fochx.2026.104742 (PMC13769120; doi:10.1016/j.fochx.2026.104742)
Supplement: Supplementary file 8 [file mmc8.docx]

**Table S5.** Effects of cultivar (Wonderful and Acco), storage time (0, 30, 60, 90, 120 days) and their interaction on colour parameters and water activity (a_w_) of dried arils of pomegranates.

|  |  | ***L**** | ***a**** | ***b**** | **Chroma** | **h°** | **a_w_** |
| --- | --- | --- | --- | --- | --- | --- | --- |
| *Cultivar (A)* |  | **** | ** | **** | *** | **** | **** |
| Wonderful |  | 19.73 b | 19.12 b | 8.17 b | 20.82 b | 23.04 b | 0.45 a |
| Acco |  | 23.27 a | 20.32 a | 10.50 a | 22.90 a | 27.26 a | 0.37 b |
| *Storage time (B)* |  | ns | ns | ns | ns | ns | ** |
| 0 |  | 21.88 | 19.69 | 9.80 | 22.03 | 26.02 | 0.38 c |
| 30 |  | 21.22 | 20.62 | 9.76 | 22.86 | 25.10 | 0.39 c |
| 60 |  | 21.07 | 18.88 | 8.82 | 20.88 | 24.85 | 0.41 bc |
| 90 |  | 21.94 | 19.91 | 9.45 | 22.07 | 25.43 | 0.43 ab |
| 120 |  | 21.40 | 19.50 | 8.83 | 21.43 | 24.35 | 0.44 a |
| AxB |  | ns | ** | ** | ** | ns | ns |
| Results are given as mean values of 30 samples (3 replicates x 2 cultivar x 5 storage time). Different letters within the same row indicate significant differences among pomegranate cultivars according to the LSD post hoc test (*p* < 0.05). The level of statistical significance is reported as follows: ns = not significant; * = *p* < 0.05; ** = *p* < 0.01; *** = p < 0.001; **** = p < 0.0001. | | | | | | | |
