# Supplementary material for: Sensory and biochemical characterization of a snack based on dried pomegranate from two cultivars during shelf-life under modified atmosphere packaging
Source: Food Chem X. 2026 Nov 2;40:104742. doi: 10.1016/j.fochx.2026.104742 (PMC13769120; doi:10.1016/j.fochx.2026.104742)
Supplement: Supplementary file 9 [file mmc9.docx]

**Table S6.** Effects of cultivar (Wonderful and Acco), storage time (0, 30, 60, 90, 120 days) and their interaction on sensory attributes of dried arils of pomegranates.

|  |  | **Visual appearence** | **Characteristics smell** | **Dislike smell** | **Taste** | **Crunchiness** | **Chewiness** | **Overall Quality** |
| --- | --- | --- | --- | --- | --- | --- | --- | --- |
| *Cultivar (A)* |  | ns | ** | ns | * | *** | ns | ns |
| Wonderful |  | 3.64 | 2.97 a | 4.56 | 3.74 a | 3.89 a | 2.74 | 3.65 |
| Acco |  | 3.58 | 2.16 b | 4.26 | 3.31 b | 3.34 b | 3.29 | 3.39 |
| *Storage time (B)* |  | ns | ns | *** | ns | ** | ns | ns |
| 0 |  | 3.76 | 2.69 | 4.68 ab | 4.04 | 4.09 a | 2.98 | 3.87 |
| 30 |  | 3.84 | 2.81 | 4.83 a | 3.21 | 3.82 ab | 3.25 | 3.44 |
| 60 |  | 3.58 | 2.79 | 4.69 ab | 3.66 | 3.55 bc | 3.62 | 3.63 |
| 90 |  | 3.51 | 2.08 | 4.28 b | 3.34 | 3.31 c | 2.90 | 3.45 |
| 120 |  | 3.37 | 2.47 | 3.57 c | 3.38 | 3.32 c | 2.32 | 3.22 |
| AxB |  | ns | * | **** | * | ns | ns | ns |
| Results are given as mean values of 50 samples (5 replicates x 2 cultivar x 5 storage time). Different letters within the same row indicate significant differences among pomegranate cultivars according to the LSD post hoc test (*p* < 0.05). The level of statistical significance is reported as follows: ns = not significant; * = *p* < 0.05; ** = *p* < 0.01; *** = p < 0.001; **** = p < 0.0001. | | | | | | | | |
